# Gsslasso Cox: a Bayesian hierarchical model for predicting survival and detecting associated genes by incorporating pathway information

**DOI:** 10.1186/s12859-019-2656-1

**Published:** 2019-02-27

**Authors:** Zaixiang Tang, Shufeng Lei, Xinyan Zhang, Zixuan Yi, Boyi Guo, Jake Y. Chen, Yueping Shen, Nengjun Yi

**Affiliations:** 1 Department of Biostatistics, School of Public Health, Medical College of Soochow University, University of Alabama at Birmingham, Suzhou, 215123 China; 2 Jiangsu Key Laboratory of Preventive and Translational Medicine for Geriatric Diseases, Medical College of Soochow University, Suzhou, 215123 China; 3 Department of Biostatistics, Jiann-Ping Hsu College of Public Health, Georgia Southern University, Statesboro, GA 30458 USA; 4 Eastern Virginia Medical School, Norfork, VA 23507 USA; 5 Department of Biostatistics, School of Public Health, University of Alabama at Birmingham, Birmingham, AL 35294-0022 USA; 6 Informatics Institute, School of Medicine, University of Alabama at Birmingham, Birmingham, AL 35294 USA

**Keywords:** Cox survival models, Grouped predictors, Hierarchical modeling, Lasso, Pathway, Spike-and-slab prior

## Abstract

**Background:**

Group structures among genes encoded in functional relationships or biological pathways are valuable and unique features in large-scale molecular data for survival analysis. However, most of previous approaches for molecular data analysis ignore such group structures. It is desirable to develop powerful analytic methods for incorporating valuable pathway information for predicting disease survival outcomes and detecting associated genes.

**Results:**

We here propose a Bayesian hierarchical Cox survival model, called the group spike-and-slab lasso Cox (gsslasso Cox), for predicting disease survival outcomes and detecting associated genes by incorporating group structures of biological pathways. Our hierarchical model employs a novel prior on the coefficients of genes, i.e., the group spike-and-slab double-exponential distribution, to integrate group structures and to adaptively shrink the effects of genes. We have developed a fast and stable deterministic algorithm to fit the proposed models. We performed extensive simulation studies to assess the model fitting properties and the prognostic performance of the proposed method, and also applied our method to analyze three cancer data sets.

**Conclusions:**

Both the theoretical and empirical studies show that the proposed method can induce weaker shrinkage on predictors in an active pathway, thereby incorporating the biological similarity of genes within a same pathway into the hierarchical modeling. Compared with several existing methods, the proposed method can more accurately estimate gene effects and can better predict survival outcomes. For the three cancer data sets, the results show that the proposed method generates more powerful models for survival prediction and detecting associated genes. The method has been implemented in a freely available R package BhGLM at https://github.com/nyiuab/BhGLM.

**Electronic supplementary material:**

The online version of this article (10.1186/s12859-019-2656-1) contains supplementary material, which is available to authorized users.

## Background

Survival prediction from high-dimensional molecular data is an active topic in the fields of genomics and precision medicine, especially for various cancer studies. Large-scale omics data provide extraordinary opportunities for detecting biomarkers and building accurate prognostic and predictive models. However, such high-dimensional data also introduce statistical and computational challenges. Tibshirani [1, 2] has proposed a novel penalized method, lasso, for variable selection in high-dimensional data, which has attracted considerable attention in modern statistical research. Thereafter, several penalized methods were developed, like minimax concave penalty (MCP) method by Zhang [3, 4], smoothly clipped absolute deviation (SCAD) penalty method by Fan and Li [5]. These penalization approaches have been widely applied for disease prediction and prognosis using large-scale molecular data [6–11].

Furthermore, the group structures among molecular variables was noticed in analysis. For example, genes can be grouped into known biological pathways or functionally similar sets. Genes within a same biological pathway may be biologically related and statistically correlated. Incorporating such biological grouping information into statistical modeling can improve the interpretability and efficiency of the models. Several penalization methods have been proposed had been proposed to utilize the grouping information, such as group Lasso method [12], sparse group lasso (SGL) [13, 14]. group bridge [15], composite MCP [16], composite absolute penalty method [17], group exponential Lasso [18], group variable selection via convex log-exp-sum penalty method [19], and doubly sparse approach for group variable selection [20]. Some of these methods perform group level selection, including or excluding an entire group of variables. Others can perform bi-level selection, achieving sparsity within each group. Huang et al. [21] and Ogutu et al. [22] reviewed these penalization methods in prediction and highlighted some issues for further study.

Ročková and George [23, 24] recently proposed a new Bayesian approach, called the spike-and-slab lasso, for high-dimensional normal linear models using the spike-and-slab mixture double-exponential prior distribution. Based on the Bayesian framework, we have recently incorporated the spike-and-slab mixture double-exponential prior into generalized linear models (GLMs) and Cox survival models, and developed the spike-and-slab lasso GLMs and Cox models for predicting disease outcomes and detecting associated genes [25, 26]. More recently, we have developed the group spike-and-slab lasso GLMs [27] to incorporate biological pathways.

In this article, we aim to develop the group spike-and-slab lasso Cox model (gsslasso Cox) for predicting disease survival outcomes and detecting associated genes by incorporating biological pathway information. An efficient algorithm was proposed to fit the group spike-and-slab lasso Cox model by integrating Expectation-Maximization (EM) steps into the extremely fast cyclic coordinate descent algorithm. The novelty is incorporating group or biological pathway information into the spike-and-slab lasso Cox model for predicting disease survival outcomes and detecting associated genes. The performance of the proposed method was evaluated via extensive simulations and comparing with several commonly used methods. The proposed procedure was also applied to three cancer data sets with thousands of gene expression values and their pathways information. Our results show that the proposed method not only generates powerful prognostic models for survival prediction, but also excels at detecting associated genes.

## Methods

### The group spike-and-slab lasso Cox models

In Cox survival model, variables *y*_*i*_ = (*t*_*i*_, *d*_*i*_) for each individual is the survival outcome. The censoring indicator *d*_*i*_ takes 1 if the observed survival time *t*_*i*_ for individual *i* is uncensored. The *d*_*i*_ takes 0 if it is censored. For individual *i*, the true survival time is assumed by *T*_*i*_. Therefore, when *T*_*i*_ = *t*_*i*_, *d*_*i*_ = 1, whereas when *T*_*i*_ > *t*_*i*_, *d*_*i*_ = 0. The predictor variables include numerous molecular predictors (e.g., gene expression) and some relevant demographic/clinical covariates. Assume that the predictors can be organized into *G* groups (e.g., biological pathways) based on existing biological knowledge. It should be indicated that the group could overlap each other. For example, one or some genes can belong to two or more biological pathway. Following the idea of overlap group lasso [28–31], we performed a restructure step by replicating a variable in whatever group it appears to expand the vector of predictors.

In Cox proportional hazards model, it usually assumes that the hazard function of survival time *T* takes the form [32, 33]:

1 $$ h\left(t|X\right)={h}_0(t)\exp \left( X\beta \right) $$

where the baseline hazard function *h*_0_(*t*) is unspecified, *X* and *β* are the vectors of explanatory variables and coefficients, respectively, and *Xβ* is the linear predictor or called the prognostic index.

Fitting classical Cox models is to estimate *β* by maximizing the partial log-likelihood [34]:

2 $$ pl\left(\beta \right)=\sum \limits_{i=1}^n{d}_i\log \left(\exp \left({X}_i\beta \right)/\sum \limits_{i\hbox{'}\in R\left({t}_i\right)}\exp \left({X}_{i\hbox{'}}\beta \right)\right) $$

where *R*(*t*_*i*_) is the risk set at time *t*_*i*_. In the presence of ties, the partial log-likelihood can be approximated by the Breslow or the Efron methods [35, 36]. The standard algorithm for maximizing the partial log-likelihood is the Newton-Raphson algorithm [32, 37].

For high dimensional and/or correlated data, the classical model fitting is often unreliable. The problem can be solved by using Bayesian hierarchical modeling or penalization approaches [31, 38, 39]. We here propose a Bayesian hierarchical modeling approach, which allows us to simultaneously analyze numerous predictors and more importantly provides an efficient way to incorporate group information. Our hierarchical Cox models employ the spike-and-slab mixture double-exponential (de) prior on the coefficients:

3 $$ {\beta}_j\mid {\gamma}_j,{s}_0,{s}_1\sim \mathrm{de}\left(0,\left(1-{\gamma}_j\right){s}_0+{\gamma}_j{s}_1\right)=\frac{1}{\left(1-{\gamma}_j\right){s}_0+{\gamma}_j{s}_1}\exp \left(-\frac{\left|{\beta}_j\right|}{\left(1-{\gamma}_j\right){s}_0+{\gamma}_j{s}_1}\right) $$

where, *s*_0_ and *s*_1_ are the preset scale parameters, which are small and relatively large (0 < *s*_0_ < *s*_1_), inducing strong or weak shrinkage on *β*_*j*_, respectively. *γ*_*j*_ is the indicator variable: *γ*_*j*_ = 1 or 0. Equivalently, this prior can be expressed as (1 - *γ*_*j*_) de(0, *s*_0_) + *γ*_*j*_ de(0, *s*_1_), a mixture of the shrinkage prior de(0, *s*_0_) and the weakly informative prior de(0, *s*_1_), which are the spike and slab components of the prior distribution, respectively.

We incorporate the group structure by proposing a group-specific Berllouli distribution for the indicator variables. For predictors in group *g*, the indicator variables are assumed to follow the Berllouli distribution with the group-specific probability *θ*_*g*_:

4 $$ {\gamma}_j\mid {\theta}_g\sim \mathrm{Bin}\left({\gamma}_j|1,{\theta}_g\right)={\theta}_g^{\gamma_j}{\left(1-{\theta}_g\right)}^{1-{\gamma}_j} $$

If group *g* includes important predictors, the parameter *θ*_*g*_ will be estimated to be relatively large, implying other predictors in the group more likely to be important. Therefore, the group-specific Berllouli prior plays a role on incorporating the biological similarity of genes within a same pathway into the hierarchical model. For the probability parameters, we adopt a beta prior, *θ*_*g*_~beta(*a*, *b*), setting *a* = *b* = 1 yielding the uniform hyper prior *θ*_*g*_~*U*(0, 1) that will be used in later sections to illustrate our method. Hereafter, the above hierarchical Cox models are referred to as the group spike-and-slab lasso Cox model.

### The EM coordinate descent algorithm

We have developed a fast deterministic algorithm, called the EM coordinate descent algorithm to fit the spike-and-slab lasso Cox models by estimating the posterior modes of the parameters [26]. The EM coordinate descent algorithm incorporates EM steps into the cyclic coordinate descent procedure for fitting the penalized lasso Cox models, and has been shown to be fast and efficient for analyzing high-dimensional survival data [26]. We here extend the EM coordinate descent algorithm to fit the group spike-and-slab lasso Cox models. We derive the algorithm based on the log joint posterior density of the parameters*ϑ* = (*β*, *γ*, *θ*):

5 $$ {\displaystyle \begin{array}{l}\log p\left(\beta, \gamma, \theta |t,d\right)\propto \log p\left(t,d|\beta, {h}_0\right)+{\sum}_{j=1}^J\log p\left({\beta}_j|{S}_j\right)\\ {}+{\sum}_{j=1}^J\log p\left({\gamma}_j|{\theta}_g\right)+{\sum}_{g=1}^G\log p\left({\theta}_g\right)\end{array}} $$

The log-likelihood function, log*p*(*t*, *d*| *β*, *h*_0_), is proportional to the partial log-likelihood *pl*(*β*) defined in Eq. (2) or the Breslow or the Efron approximation in the presence of ties [35, 36], if the baseline hazard function *h*_0_ is replaced by the Breslow estimator [37, 40]. Therefore, the log joint posterior density can be expressed as

6 $$ {\displaystyle \begin{array}{l}\log p\left(\beta, \gamma, \theta |t,d\right)\propto pl\left(\beta \right)-{\sum}_{j=1}^J{S}_j^{-1}\left|{\beta}_j\right|+{\sum}_{j=1}^J\left({\gamma}_j\log {\theta}_g+\left(1-{\gamma}_j\right)\log \left(1-{\theta}_g\right)\right)\\ {}\kern8.25em +{\sum}_{g=1}^G\left(\left(a-1\right)\log {\theta}_g+\left(b-1\right)\log \left(1-{\theta}_g\right)\right)\end{array}} $$

where *pl*(*β*) is the partial likelihood described in (2), and *S*_*j*_ = (1 − *γ*_*j*_)*s*_0_ + *γ*_*j*_*s*_1_.

In EM coordinate decent algorithm, the indicator variables *γ*_*j*_ were treated the as ‘missing values’. The parameters (*β*, *θ*) were estimated by averaging the missing values over their posterior distributions. For the E-step, the expectation of the log joint posterior density was calculated with respect to the conditional posterior distributions of the missing data. For predictors in group *g*, the conditional posterior expectation of the indicator variable *γ*_*j*_ can be derived as

7 $$ {\displaystyle \begin{array}{l}{p}_j^g=p\left({\gamma}_j=1|{\beta}_j,{\theta}_g,t,d\right)\\ {}=\frac{p\left({\beta}_j|{\gamma}_j=1,{s}_1\right)p\left({\gamma}_j=1|{\theta}_g\right)}{p\left({\beta}_j|{\gamma}_j=0,{s}_0\right)p\left({\gamma}_j=0|{\theta}_g\right)+p\left({\beta}_j|{\gamma}_j=1,{s}_1\right)p\left({\gamma}_j=1|{\theta}_g\right)}\end{array}} $$

where *p*(*γ*_*j*_ = 1| *θ*_*g*_) = *θ*_*g*_, *p*(*γ*_*j*_ = 0| *θ*_*g*_) = 1 − *θ*_*g*_, *p*(*β*_*j*_| *γ*_*j*_ = 1, *s*_1_) = de(*β*_*j*_| 0, *s*_1_) and *p*(*β*_*j*_| *γ*_*j*_ = 0, *s*_0_) = de(*β*_*j*_| 0, *s*_0_). Therefore, the conditional posterior expectation of ${S}_j^{-1}$ can be obtained by

8 $$ E\left({S}_j^{-1}|{\beta}_j\right)=E\left(\frac{1}{\left(1-{\gamma}_j\right){s}_0+{\gamma}_j{s}_1}|{\beta}_j\right)=\frac{1-{p}_j^g}{s_0}+\frac{p_j^g}{s_1} $$

From Eqs. (7) and (8), we can see that the estimates of *p*_*j*_ and *S*_*j*_ are larger for larger coefficients*β*_*j*_, leading to different shrinkage for different coefficients.

For the M-step, parameters (*β*, *θ*) were updated by maximizing the posterior expectation of the log joint posterior density with *γ*_*j*_ and ${S}_j^{-1}$ replaced by their conditional posterior expectations. From the log joint posterior density, we can see that *β* and *θ* can be updated separately, because the coefficients *β* are only involved in$pl\left(\beta \right)-{\sum}_{j=1}^J{S}_j^{-1}\left|{\beta}_j\right|$ and the probability parameter *θ* is only in ${\sum}_{j=1}^J\left({\gamma}_j\log {\theta}_g+\left(1-{\gamma}_j\right)\log \left(1-{\theta}_g\right)\right)+{\sum}_{g=1}^G\left(\left(a-1\right)\log {\theta}_g+\left(b-1\right)\log \left(1-{\theta}_g\right)\right)$. Therefore, the coefficients *β* are updated by maximizing the expression:

9 $$ {Q}_1\left(\beta \right)= pl\left(\beta \right)-{\sum}_{j=1}^J{\widehat{S}}_j^{-1}\left|{\beta}_j\right| $$

where ${\widehat{S}}_j^{-1}$ is the conditional posterior expectation of ${S}_j^{-1}$ as derived above. Given the scale parameters *S*_*j*_, the term ${\sum}_{j=1}^J{\widehat{S}}_j^{-1}\left|{\beta}_j\right|$ serves as the *L*_1_ lasso penalty with ${\widehat{S}}_j^{-1}$ as the penalty factors, and thus the coefficients can be updated by maximizing *Q*_1_(*β*) using the cyclic coordinate decent algorithm, which is extremely fast and can estimate some coefficients exactly to zero [31, 41]. The probability parameters {*θ*_*g*_} are updated by maximizing the expression:

10 $$ {Q}_2\left(\theta \right)=\sum \limits_{j=1}^J\left[{p}_j^g\log {\theta}_g+\left(1-{p}_j^g\right)\log \left(1-{\theta}_g\right)\right]+{\sum}_{g=1}^G\left(\left(a-1\right)\log {\theta}_g+\left(b-1\right)\log \left(1-{\theta}_g\right)\right) $$

We can easily obtain:

11 $$ {\theta}_g=\frac{\sum \limits_{j\in g}{p}_j^g+a-1}{J_g+a+b-2} $$

where *J*_*g*_ is the number of predictors belonging to group *g*.

Totally, the framework of the proposed EM coordinate decent algorithm was summarized as follows:

1. Choose a starting value for *β*^0^, and ${\theta}_g^0$. For example, we can initialize *β*^0^ = 0, and ${\theta}_g^0=0.5$.
2. For t = 1, 2, 3, …,

E-step: Update *γ*_*j*_ and ${S}_j^{-1}$ by their conditional posterior expectations.

M-step:

- Update *β* using the cyclic coordinate decent algorithm;
- Update (*θ*_1_, ⋯, *θ*_*G*_) by Eq. (11).

We assess convergence by the criterion: ∣*d*^(*t*)^ − *d*^(*t* − 1)^ ∣ /(0.1−| *d*^(*t*)^| ) < *ε*, where *d*^(*t*)^ =  − 2*pl*(*β*^(*t*)^) is the estimate of deviance at the *t*^th^ iteration, and *ε* is a small value (say 10^− 5^).

### Evaluation of predictive performance

We can use several ways to measure the performance of a fitted group lasso Cox model, including the partial log-likelihood (PL), the concordance index (C-index), the survival curves, and the survival prediction error [37]. The partial log-likelihood function measures the overall quality of a fitted Cox model, and thus is usually used to choose an optimal model [37, 41, 42]. The standard way to evaluate the performance of a model is to fit the model using a data set and then calculate the above measures with independent data. A variant of cross-validation [31, 43], called pre-validation method was used in the present study to evaluate the performance. The data was randomly split to *K* subsets of roughly the same size. The (*K* – 1) subsets was used to fit a hierarchical Cox model. The estimate of coefficients denoted as ${\widehat{\beta}}^{\left(-k\right)}$ from the data excluding the *k*-th subset. The prognostic indices ${\widehat{\eta}}_{(k)}={X}_{(k)}{\widehat{\beta}}^{\left(-k\right)}$, called the cross-validated or pre-validated prognostic index, were calculated for all individuals in the *k*-th subset of the data. Cross-validated prognostic indices ${\widehat{\eta}}_i$ for all individuals can be calculated by cycling through all the *K* parts. Then, (${t}_i,{d}_i,{\widehat{\eta}}_i$) was used to compute the several measures described above. We can see that the cross-validated prognostic value for each patient is derived independently of the observed response of the patient. Therefore, the ‘pre-validated’ dataset (${t}_i,{d}_i,{\widehat{\eta}}_i$) can essentially be treated as a ‘new dataset’. This procedure provides valid assessment of the predictive performance of the model [31, 43].

Moreover, we also use an alternative way to evaluate the partial log-likelihood, i.e., the so-called cross-validated partial likelihood (CVPL), defined as [37, 41, 42].

12 $$ CVPL={\sum}_{k=1}^K\left[ pl\left({\widehat{\beta}}_{\left(-k\right)}\right)-{pl}_{\left(-k\right)}\left({\widehat{\beta}}_{\left(-k\right)}\right)\right] $$

where ${\widehat{\beta}}_{\left(-k\right)}$ is the estimate of *β* from all the data except the *k*-th part, $pl\left({\widehat{\beta}}_{\left(-k\right)}\right)$ is the partial likelihood of all the data points and ${pl}_{\left(-k\right)}\left({\widehat{\beta}}_{\left(-k\right)}\right)$ is the partial likelihood excluding part *k* of the data. By subtracting the log-partial likelihood evaluated on the non-left out data from that evaluated on the full data, we can make efficient use of the death times of the left out data in relation to the death times of all the data.

### Selecting optimal scale values

The spike-and-slab double-exponential prior requires two preset scale parameters (*s*_0_, *s*_1_). Following the previous studies [24–26], we set the slab scale *s*_1_ to be relatively large (e.g., 1), and consider a sequence of *L* decreasing values {${s}_0^l$}: ${s}_1>{s}_0^1>{s}_0^2>\cdots >{s}_0^L>0$, for the spike scale *s*_0_. We then fit *L* models with scales {$\left({s}_0^l,{s}_1\right);l=1,\cdots, L$} and select an optimal model using the method described above. This procedure is similar to the lasso implemented in the widely-used R package glmnet, which quickly fits the lasso Cox models over a grid of values of *λ* covering its entire range, giving a sequence of models for users to choose from [31, 41].

### Implementation and software package

We have incorporated the method proposed in this study into the function bmlasso() in our R package BhGLM [44]. The package BhGLM also includes several other functions for summarizing and evaluating the predictive performance, like summary.bh, cv.bh predict.bh. The function in the package is very fast, usually taking several minutes for fitting and evaluating a model with thousands of variables. The package BhGLM is freely available from https://github.com/nyiuab/BhGLM.

## Simulation study and real data analysis

### Simulation studies

We assessed the proposed approach by extensive simulations, and compared with the lasso implemented in the R package glmnet and several penalization methods that can incorporate group information, including sparse group lasso (SGL) in the R package SGL, overlap group lasso (grlasso), overlap group MCP (grMCP), overlap group SCAD (grSCAD), and overlap group composite MCP (cMCP) in the R package grpregOverlap [45]. Our simulation method was similar to our previous work [26, 27]. We considered five simulation scenarios with different complexities, including non-overlap or overlap groups, group sizes, number of non-null groups, and correlation coefficients (*r*) (Table 1). In simulation scenario 2–5, overlap structures were considered. To handle the overlap structures, we duplicated overlapping predictors into groups that predictors belong to [28, 30]. In each scenario, we simulated two data sets, and used the first one as the training data to fit the models and the second one as the test data to evaluate the predictive values. We replicated the simulation 100 times and summarized the results over these replicates. In simulation scenario 6, we vary the effect size of the non-zero coefficient *β*_5_, from − 2 to 2. Other simulation setting are the same with scenario 2. The purpose of this simulation is to see the profile of prior scale along with varying effect size.

**Table 1:** The preset non-zero predictors and their assumed effect values of the different simulation scenarios

Simulation scenarios	Group, non-zero predictors and effect size							
1 non-overlap group								
Group	group1			group5			group20	
predictors	{*x*_5_	*x*_20_	*x*_40_}	{*x*_210_	*x*_220_	*x*_240_}	{*x*_975_	*x*_995_}
2 overlap group								
Group	group1			group5			group20	
predictors	{*x*_5_	*x*_20_	*x*_40_}	{*x*_210_	*x*_220_	*x*_240_}	{*x*_975_	*x*_995_}
3 varying group size (4/20/50)								
Group	group1				group11			
predictors	{*x*_1_	*x*_2_	*x*_3_	*x*_4_}	{*x*_501_	*x*_502_	*x*_503_	*x*_504_}
4 varying number of non-null groups (8/3/1)								
Group	group1	group2	group7	group8	group11	group12	group19	group20
predictors	{*x*_5_}	{*x*_55_}	{*x*_305_}	{*x*_355_}	{*x*_505_}	{*x*_555_}	{*x*_905_}	{*x*_955_}
Group	group1			group8			group20	
predictors	{*x*_5_	*x*_15_	*x*_25_}	{*x*_355_	*x*_365_	*x*_375_}	{*x*_905_	*x*_915_}
Group	group1							
predictors	{*x*_5_	*x*_10_	*x*_15_	*x*_20_	*x*_25_	*x*_30_	*x*_35_	*x*_40_}
5 varying correlation within group (*r = 0.0/0.5/0.7*)								
Group	group1			group5			group20	
predictors	{*x*_5_	*x*_20_	*x*_40_}	{*x*_210_	*x*_220_	*x*_240_}	{*x*_975_	*x*_995_}
Effect size for above simulation scenarios								
	0.8	−0.7	1.0	−0.9	−0.8	0.9	−1.0	0.7
6 varying effect size								
Group	group1			group5			group20	
predictors	{*x*_5_	*x*_20_	*x*_40_}	{*x*_210_	*x*_220_	*x*_240_}	{*x*_975_	*x*_995_}
Effect size for scenario 6								
	(−2, 2)	−0.7	1.0	−0.9	−0.8	0.9	−1.0	0.7

Note:{} quotes the predictors within a group.

Each simulated dataset included *n* = 500 observations, with a censored survival response *y*_*i*_ and a vector of *m* = 1000 continuous predictors*X*_*i*_ = (*x*_*i*1_, …, *x*_*im*_). We assumed 20 groups. Each group included about 50 predictors. For example, group 1 and 2 included variables (*x*_1_, …, *x*_50_) and (*x*_51_, …, *x*_100_), respectively. The vector *X*_*i*_ was randomly sampled from multivariate normal distribution*N*_1000_(0, Σ), where the covariance matrix Σ was set to account for varied grouped correlation and overlapped structures under different simulation scenarios. We simulated several scenarios. The predictors were assumed to be correlated each other with in group and those predictors in different groups were assumed to be independent. The correlation coefficient r was generally set to be 0.5.

 To simulate the censored survival response, following the method of Simon [41], we generated the “true” survival time *T*_*i*_ for each individual from the exponential distribution: ${T}_i\sim \mathrm{Expon}\left(\exp \left({\sum}_{j=1}^m{x}_{ij}{\beta}_j\right)\right)$ and the censoring time *C*_*i*_ for each individual from the exponential distribution: *C*_*i*_~Expon(exp(*r*_*i*_)), where *r*_*i*_ were randomly sampled from a standard normal distribution. The observed censored survival time *t*_*i*_ was set to be the minimum of the “true” survival and censoring times, *t*_*i*_ = min(*T*_*i*_, *C*_*i*_), and the censoring indicator *d*_*i*_ was set to be 1 if *C*_*i*_ > *T*_*i*_ and 0 otherwise. Our simulation scenarios resulted in different censoring ratios, but generally below 50%. For all the scenarios, we set eight coefficients to be non-zero and the others to be zero.

#### Scenario 1: Non-overlap group

In this scenario, each group is independent. There was no any overlap among groups. Eight non-zero predictors{*x*_5_, *x*_20_, *x*_40_},  {*x*_210_,  *x*_220_,  *x*_240_},  {*x*_975_,  *x*_995_} were simulated to be included into three groups, group 1, 5, and 20 (Table 1). The group sizes is 50, including 50 predictors, presented as below:

**Table:** 

Group ID:	1	2	…	5	…	19	20
Group setting:	*x*_1_ − *x*_50_	*x*_51_ − *x*_100_		*x*_201_ − *x*_250_		*x*_901_ − *x*_950_	*x*_951_ − *x*_1000_

#### Scenario 2: Overlap grouping

In this scenario, overlapped grouping structure was considered. Only the last group is independent. For example, for group 1 and group 2, there were five predictors (*x*_46_, *x*_47_, *x*_48_, *x*_49_, *x*_50_) belong to two groups. The setting for eight non-zero predictors and their effect sizes are the same with scenario 1. The group sizes is still 50. The overlap structure are presented below:

**Table:** 

Group ID:	1	2	3	…	19	20
Group setting:	*x*_1_ *− x*_50_	*x*_46_ − *x*_100_	*x*_96_ − *x*_150_		*x*_896_ − *x*_950_	*x*_951_ − *x*_1000_

#### Scenario 3: Varying group sizes

Group size means the number of predictors included in a group. A big group size means the group included relative more predictors. The group size may affect the model fitting. In this scenario, we assumed two groups, group 1 and 11, including non-zero predictors, {*x*_1_, *x*_2_, *x*_3_, *x*_4_} and {*x*_501_, *x*_502_, *x*_503_, *x*_504_}, respectively. Other simulation setting are similar with scenario 2. To investigate the group size effect on model fitting, we simulated different group size as below:

1. only four non-zero predictors included in group 1 and 11:

**Table:** 

Group ID:	1	2	3	…	11	12	…	19	20
Group setting:	*x*_1_ - *x*_4_	*x*_5_ - *x*_100_	*x*_96_ - *x*_150_		*x*_501_ - *x*_504_	*x*_505_ - *x*_600_		*x*_896_ - *x*_950_	*x*_951_ - *x*_1000_

(2). 20 predictors included in group 1 and 11:

**Table:** 

Group ID:	1	2	3	…	11	12	…	19	20
Group setting:	*x*_1_ - *x*_20_	*x*_21_ - *x*_100_	*x*_96_ - *x*_150_		*x*_501_ - *x*_520_	*x*_521_ -*x*_600_		*x*_896_ - *x*_950_	*x*_951_ - *x*_1000_

(3). 50 predictors included in group 1 and 11:

**Table:** 

Group ID:	1	2	3	…	11	12	…	19	20
Group setting:	*x*_1_ - *x*_50_	*x*_46_ - *x*_100_	*x*_96_ - *x*_150_		*x*_501_ - *x*_550_	*x*_546_ - *x*_600_		*x*_896_ - *x*_950_	*x*_951_ - *x*_1000_

#### Scenario 4: Varying the number of non-null group

The true non-zero predictors may be included in some groups. Other zero predictors belong to other groups. These groups included non-zero predictors called non-null group. The number of non-null group may also affect the model fitting. To evaluate the group number effect, we varied the number of non-null groups, as following:

1. There are 8 non-null groups including non-zero coefficients: {*x*_5_}, {*x*_55_}, {*x*_305_}, {*x*_355_}, {*x*_505_}, {*x*_555_}, {*x*_905_}, and {*x*_955_};
2. There are 3 non-null groups including non-zero coefficients: {*x*_5_, *x*_15_, *x*_25_}, {*x*_355_, *x*_365_, *x*_375_}, and {*x*_905_, *x*_915_};
3. There is only 1 non-null group including non-zero coefficients: {*x*_5_, *x*_10_, *x*_15_, *x*_20_, *x*_25_, *x*_30_, *x*_35_, *x*_40_}. The overlap settings were the same with scenario 2. The group number and effect sizes of these non-zero coefficients are shown in Table 1.

#### Scenario 5: Varying the correlation within group

To evaluate the effect of correlation within group, we set different correlation coefficients within a group: *r* = 0.0, 0.5, and 0.7. Other settings were the same with scenario 2.

#### Scenario 6: Self-adaptive shrinkage on varying the effect size

The significant feature of the proposed spike-and-slab prior is the self-adaptive shrinkage. To show this property, we performed additional simulation study based on Scenario 1. We fixed the prior scale (*s*_0_, *s*_1_) = (0.02, 1) and varied the effect size of the first simulated non-zero predictor (*x*_5_) from (− 2, 2). We recorded the scale parameters for this non-zero predictor (*x*_5_) and nearby zero effect predictor (*x*_6_), and non-zero predictor (*x*_20_) with the simulated effect size − 0.7. These three predictors belong to the same group.

### Real data analysis

We applied the proposed gsslasso Cox model to analyze three real datasets, ovarian cancer (OV), lung adenocarcinoma (LUAD), and breast cancer. The whole genome expression data were downloaded from The Cancer Genome Atlas (TCGA, http://cancergenome.nih.gov/) (updated at June 2017). We firstly clean the data to get the clear survival information and potential genes involved in further analysis. The details of the three datasets and clean procedure are described below paragraphs. Secondly, several genome annotation tools were used to construct the pathways information. All the genes were mapped to KEGG pathways by using *R/bioconductor* packages: *mygene*, clusterProfiler *and AnnotationDbi* [46]. The R/Bioconductor mygene package was used to convert gene names to gene ENTREZ ID. The clusterProfiler package was used to get pathway/group information for genes, by loading the gene ENTREZ ID. AnnotationDbi was used primarily to create mapping objects that allow easy access from R to underlying annotation databases, like KEGG in the present study. By using these packages, we mapped the genes into pathways, and got group structure information for further analysis. Only the gene included in pathways were used in further analysis. Thirdly, the proposed method and several penalization approaches used in above simulation study were applied to analyze the survival data with thousands of genes and pathway/group information. We performed 10-fold cross-validation with 10 replicates to evaluate the predictive values of the several models. After model fitting, the non-zero parameters were the detected genes.

#### TCGA ovarian cancer dataset (mRNA sequencing data)

This dataset contains mRNA expression data and relevant clinical outcome for ovarian cancer (OV) from TCGA. The raw dataset includes 304 patients and 20,503 genes after removing the duplication and unknown gene names. The raw clinical data included 586 patients. We cleaned the clinical survival data from several clinical files, and obtained 582 patients with clear survival information. We merged the individuals both with gene expression data and survival information, and obtained 304 patients with 20,503 genes for further analysis. First, we filtered the genes with expressions values less than 10. Then, genes with more than 30% of zero expression values in the dataset were removed. Furthermore, we calculated the coefficient of variance (CV) of expression values for each gene, and kept the genes with CV of larger than 20% quantile. After these steps, 304 patients with 14,265 genes were included in our analysis. The censoring ratio was 39.5%.We mapped these genes to 271 pathways including 4260 genes.

#### TCGA lung adenocarcinoma dataset (mRNA sequencing data)

The raw expression data contains 578 patients and 20,530 genes. After removing the duplication and unknown gene names, there are 516 patients with 20,501 used for further analysis. The raw clinical data included 521 patients. We cleaned the clinical data with clear survival records, and included 497 patients in our analysis. We then merged the clinical data and expression data, and obtained 491 patients for with 20,501 genes for quality control. Similar with the steps for ovarian cancer dataset, we filtered the genes with expressions values less than 10. Then, we removed genes with more than 30% of zero expression values in the dataset. Furthermore, we calculated the coefficient of variance (CV) of expression values for each gene, and kept the genes with CV of larger than 20% quantile. After these steps, 491 patients with 14,143 genes were included in our analysis. The censoring ratio was 68.4%. We mapped these genes to 274 pathways including 4266 genes.

#### TCGA breast cancer dataset (mRNA sequencing data)

The raw expression data contains 1220 patients and 20,530 genes. After removing the duplication and unknown gene names, there are 1097 patients with 20,503 used for further analysis. The raw clinical data included 1097 patients. We cleaned the clinical data with clear survival records, and included 1084 patients in our analysis. We then merged the clinical data and expression data, and obtained 1082 patients for with 20,503 genes for quality control. The same steps used here for breast cancer dataset, we filtered the genes with expressions values less than 10, and removed genes with more than 30% of zero expression values in the dataset. Furthermore, we calculated the coefficient of variance (CV) of expression values for each gene, and kept the genes with CV of larger than 20% quantile. After these steps, 1082 patients with 14,077 genes were included in our analysis. The censoring ratio was 86.0%. We mapped these genes to 275 pathways including 4385 genes.

## Results

### Simulation results

#### Predictive performance

Tables 2 and 3 summarizes the CVPL (cross-validated partial likelihood) and C-index in the testing data over 100 replicates for Scenarios 1–5. We observed that the group spike-and-slab lasso Cox model performed similarly with cMCP and outperformed other methods, under different simulation scenarios. These results suggested that, with complex group structures, the proposed method could perform well.

**Table 2:** Estimates of two measures over 100 replicates under simulation scenario 1 and 2

	Methods	CVPL	C-index
Scenario 1	gsslasso	− 1111.541(52.390)	0.848(0.012)
	lasso	− 1140.742(52.108)	0.836(0.013)
	grplasso	− 1198.449(53.664)	0.792(0.017)
	grMCP	− 1280.783(66.870)	0.736(0.039)
	grSCAD	− 1256.297(57.293)	0.752(0.027)
	cMCP	− 1114.934(53.278)	0.847(0.012)
	SGL	− 1167.902(72.121)	0.826(0.016)
Scenario 2	gsslasso	− 1077.398(56.949)	0.868(0.011)
	lasso	−1114.886(56.200)	0.853(0.012)
	grplasso	− 1161.058 (59.318)	0.825 (0.015)
	grMCP	− 1236.072(67.840)	0.775(0.018)
	grSCAD	− 1219.129(66.240)	0.798(0.020)
	cMCP	− 1078.363 (57.004)	0.866 (0.011)

Note: Values in the parentheses are standard deviations. “gsslasso” represents the proposed group spike-and-slab lasso cox. The slab scales, *s*_1_, are 1 in the analyses. The optimal *s*_0_ = 0.02 and *s*_0_ = 0.03 for gsslasso cox methods under scenario 1 and 2, respectively. For scenarios with overlap structures, SGL method was not used for comparison since it cannot handle overlap situation directly

**Table 3:** Estimates of two measures over 100 replicates for varying group size and varying number of non-null group under simulation scenario 3,4 and 5, respectively

scenario 3				scenario 4			scenario 5		
Group size	methods	CVPL	C-index	number of non-null group	CVPL	C-index	Correlation coefficients within group	CVPL	C-index
4/4	gsslasso	− 1130.995 (58.229)	0.829 (0.0513)	8/20	− 1090.819 (53.224)	0.875 (0.010)	*r* = 0.0	−1077.130 (57.084)	0.876 (0.009)
	lasso	−1167.319 (57.844)	0.813 (0.015)		− 1113.349 (52.438)	0.870 (0.010)		− 1104.924 (56.431)	0.870 (0.010)
	grlasso	− 1137.892 (57.414)	0.827 (0.014)		− 1266.185 (57.782)	0.746 (0.018)		− 1174.234 (57.919)	0.829 (0.014)
	grMCP	− 1131.451 (57.960)	0.829 (0.013)		− 1334.359 (58.901)	0.616 (0.029)		− 1287.124 (64.897)	0.747 (0.035)
	grSCAD	− 1132.272 (58.315)	0.829 (0.013)		− 1305.299 (58.587)	0.721 (0.025)		− 1258.988 (62.970)	0.795 (0.026)
	cMCP	−1131.483 (58.339)	0.829 (0.013)		− 1094.230 (52.983)	0.875 (0.010)		−1082.770 (57.483)	0.875 (0.010)
4/20	gsslasso	− 1149.792 (56.801)	0.830 (0.013)	3/20	− 1120.043 (62.936)	0.849 (0.013)	*r* = 0.5	− 1087.823 (56.773)	0.865 (0.011)
	lasso	− 1179.653 (56.986)	0.813 (0.014)		−1149.463 (61.507)	0.836(0.015)		− 1119.388 (56.076)	0.852 (0.013)
	grlasso	−1179.498 (55.463)	0.811 (0.013)		− 1213.466 (62.431)	0.784 (0.018)		− 1157.999 (54.642)	0.828 (0.013)
	grMCP	− 1172.856 (56.712)	0.816 (0.013)		− 1318.758 (64.886)	0.685 (0.033)		− 1226.349 (62.257)	0.778 (0.018)
	grSCAD	−1172.884 (56.852)	0.816 (0.013)		− 1278.753 (62.726)	0.756 (0.023)		− 1208.197 (63.032)	0.801 (0.018)
	cMCP	− 1150.915 (56.806)	0.827 (0.013)		− 1122.606 (62.852)	0.848(0.014)		− 1089.138 (56.817)	0.864 (0.011)
4/50	gsslasso	− 1145.155 (56.523)	0.825 (0.013)	1/20	− 1141.219 (60.329)	0.824 (0.014)	*r* = 0.7	−1113.142 (60.749)	0.852 (0.012)
	lasso	− 1176.796 (56.449)	0.810 (0.015)		−1172.768 (57.418)	0.810 (0.014)		−1130.099 (60.286)	0.834 (0.013)
	grlasso	−1208.999 (55.893)	0.782 (0.017)		− 1180.395 (58.095)	0.802 (0.017)		− 1164.874 (59.066)	0.814 (0.013)
	grMCP	− 1272.423 (73.279)	0.782 (0.082)		− 1178.416 (64.849)	0.808 (0.016)		− 1202.094 (62.653)	0.822 (0.013)
	grSCAD	− 1271.286 (58.185)	0.777 (0.018)		−1178.827 (65.082)	0.808 (0.016)		− 1195.481 (63.401)	0.852 (0.012)
	cMCP	− 1148.318 (56.896)	0.824 (0.013)		− 1147.845 (59.271)	0.821 (0.014)		− 1117.158 (61.869)	0.858 (0.013)

Notes: in scenario 3, group size “4/50” denotes that there are four none-zero coefficients embedded in a group with 50 predictors. The group size is 50. This is true for “4/20” and “4/4”. The optimal *s*_0_ = 0.02 for different group size settings. In scenario 4, “8/20” denotes that there are 8 non-null groups among 20 groups. Each non-null group includes at least one non-zero coefficients. The optimal *s*_0_ = 0.02 for the three settings. In scenario 5, the optimal *s*_0_ are 0.02, 0.03, and 0.04 for different correlation coefficients, 0.0, 0.5, and 0.7 within group, respectively. The slab scales, *s*_1_, are 1 in this scenario 3 4, and 5. Values in the parentheses are standard errors. “gsslasso” represents the proposed group spike-and-slab lasso cox

#### Accuracy of parameter estimates

To evaluate the accuracy of parameters estimation, we summarized the average numbers of non-zero coefficients and the mean absolute errors (MAE) of coefficient estimates, defined as MAE = $\sum \left|{\widehat{\beta}}_j-{\beta}_j\right|/m$, in Tables 4 and 5 for different scenarios**.** It was found that the dected number of null-zero coefficients were very close preset number 8, and the values of MAE were very small for the proposed method under different scenarios. The performances of the group spike-and-slab lasso Cox and cMCP were consistently better than the other methods for all the five scenarios, and the proposed method was slightly better than cMCP. These results suggested that the proposed method can generate lowest false positive and unbiased estimation.

**Table 4:** Average number of non-zero coefficients and mean absolute error (MAE) of coefficient estimates over 100 simulations for scenario 1 and 2

	Method	Average Number	MAE
Scenario 1	gsslasso	8.61	0.60 (0.24)
	lasso	51.99	3.77 (0.40)
	grlasso	474.80	12.43 (1.64)
	grMCP	62.00	9.30 (2.56)
	grSCAD	108.80	8.41 (1.25)
	cMCP	14.19	0.96 (0.34)
	SGL	39.79	6.25 (1.65)
Scenario 2	gsslasso	9.74	1.29 (0.84)
	lasso	53.70	4.02 (0.46)
	grlasso	502.05	12.11 (2.08)
	grMCP	57.13	8.04 (0.67)
	grSCAD	167.59	8.77 (0.93)
	cMCP	15.14	0.96 (0.33)

*: the optimal *s*_0_ = 0.02 and *s*_0_ = 0.03 for gsslasso method under scenario 1 and 2, respectively. For scenarios with overlap structures, SGL method was not used for comparison since it cannot handle overlap situation directly

**Table 5:** Average number of non-zero coefficients and mean absolute error (MAE) of coefficient estimates over 100 simulations for scenario 3, 4, and 5

scenario 3: Group size						
	4/4		4/20		4/50	
	Average Number	MAE	Average Number	MAE	Average Number	MAE
gsslasso	9.09	0.58 (0.22)	9.26	0.64 (0.29)	9.27	0.69 (0.54)
lasso	53.75	3.90 (0.41)	54.58	3.94 (0.43)	53.96	3.93 (0.44)
grlasso	270.78	2.78 (1.22)	455.15	7.06 (1.69)	509.75	10.58 (1.71)
grMCP	13.40	0.57 (0.18)	40.00	3.50 (0.54)	84.50	10.09 (2.52)
grSCAD	56.16	0.85 (0.78)	53.85	3.62 (0.74)	100.00	7.86 (1.77)
cMCP	9.42	0.64 (0.29)	14.64	0.98 (0.33)	16.85	1.05 (0.38)
scenario 4: Number of non-null groups						
	8/20		3/20		1/20	
gsslasso	8.85	0.54 (0.19)	9.12	0.56 (0.19)	9.27	0.68 (0.26)
lasso	52.87	3.58 (0.44)	53.51	3.89 (0.43)	52.49	3.90 (0.41)
grlasso	757.1	19.84 (2.51)	610.25	13.91 (2.08)	461.25	7.64 (1.71)
grMCP	83.95	7.68 (0.60)	46.00	7.49 (1.51)	50.00	4.52 (0.67)
grSCAD	410.3	10.80 (0.82)	142.30	8.04 (0.74)	55.85	4.57 (0.74)
cMCP	13.22	0.83 (0.38)	15.74	0.96 (0.35)	14.81	1.03 (0.31)
scenario 5: Correlation coefficients within group						
	*r* = 0		*r* = 0.5		*r* = 0.7	
gsslasso	9.18	0.85 (0.72)	8.90	1.07 (0.99)	8.11	3.54 (0.54)
lasso	59.10	3.42 (0.35)	52.63	4.04 (0.49)	48.82	5.25 (0.50)
grlasso	557.00	10.27 (1.25)	490.50	11.88 (1.75)	465.90	13.61 (2.45)
grMCP	61.71	7.38 (0.89)	57.40	8.14 (1.29)	53.54	9.59 (0.83)
grSCAD	148.68	7.42 (0.67)	170.61	8.78 (1.16)	194.58	11.28 (1.28)
cMCP	16.72	0.98 (0.47)	14.32	0.98 (0.37)	21.51	3.53 (0.40)

Notes: in scenario 3, group size “4/50” denotes that there are four none-zero coefficients embedded in a group with 50 predictors. The group size is 50. This is true for “4/20” and “4/4”. The optimal *s*_0_ = 0.02 for different group size settings. The slab scales, *s*_1_, are 1 in this scenario. In scenario 4 “8/20” denotes that there are 8 non-null groups among 20 groups. Each non-null group includs at least one non-zero coefficients. The optimal *s*_0_ = 0.02 for the three settings. In scenario 5, the optimal *s*_0_ are 0.02, 0.03, and 0.04 for different correlation coefficients, 0.0, 0.5, and 0.7 within group, respectively. The slab scales, *s*_1_, are 1 in this scenario 3, 4 and 5. Values in the parentheses are standard errors. “gsslasso” represents the proposed group spike-and-slab lasso cox

**Fig. 1:**
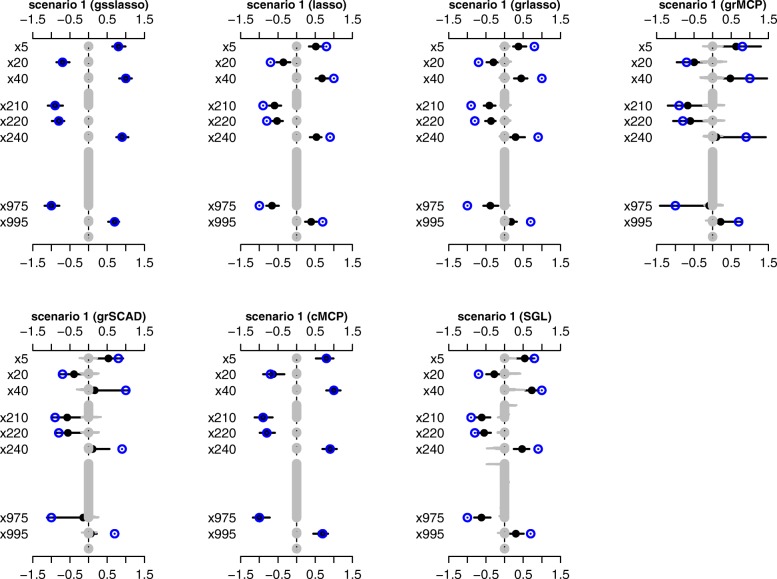
The parameter estimation averaged over 100 replicates for the group spike-and-slab lasso Cox (gsslasso), the lasso, grlasso, grMCP, grSCAD, SGL and cMCP methods for Scenario 1. Blue cycles are the simulated non-zero values. Black points and lines represent the estimated values and the interval estimates of coefficients

The estimates of coefficients from the group spike-and-slab lasso Cox and the other methods over 100 replicates are shown in Fig. 1 and Additional file 1: Figure S1, Additional file 2: Figure S2, Additional file 3: Figure S3, Additional file 4: Figure S4, Additional file 5: Figure S5, Additional file 6: Figure S6 and Additional file 7: Figure S7 for different scenarios. It can be seen that the group spike-and-slab lasso Cox method produced estimates close to the simulated values for all the coefficients. This is expected, because the spike-and-slab prior can induce weak shrinkage on larger coefficients and strong shrinkage on zero coefficients. In contrast, other methods except for cMCP, gave a strong shrinkage amount on all the coefficients and resulted in the solutions that non-zero coefficients were shrunk and underestimated compared to true values. In addition, higher false positives (grey bars) were observed, except for the group spike-and-slab lasso Cox and cMCP methods.

#### The self-adaptive shrinkage feature

**Fig. 2:**
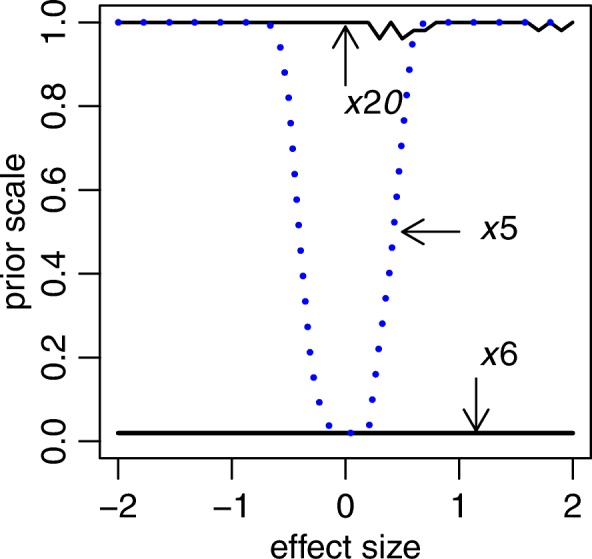
The adaptive shrinkage amount, along with the varying effect size for *x*_5_. The *x*_20_ is the none-zero coefficients with the simulated effect size − 0.7, while *x*_6_ is the nearby coefficient with simuated zero effect size

To show the self-adaptive shrinkage feature, we performed simulation 6. Figure 2 shows the adaptive shrinkage amount on non-zero coefficients *x*_5_, along with the varying effect size. It clearly shows that the proposed spike-and-slab lasso Cox model approach has self-adaptive and flexible characteristics, without affecting the nearby zero coefficient (*x*_6_) and non-zero variable (*x*_20_) belong to the same group.

### Real data analysis results

There were about one third genes were mapped to pathways for the above three real datasets. The rest genes were put together as an additional group. The detailed information of genes shared by different pathways is listed in Additional file 8: S1, S2, and S3, for ovarian cancer, lung cancer and breast cancer, respectively.

Real data analysis is to build a survival model for predicting the overall survival outcome by integrating gene expression data and pathway information. We standardized all the predictors to use a common scale for all predictors, prior to fitting the models, using the function *covariate*() function in BhGLM package. In our prior distribution, there were to preset parameters, (*s*_0_, *s*_1_). In our real data analysis, we fixed the slab scale *s*_1_ to 1, and varied the spike scale *s*_0_ values by: {*k* × 0.01; *k* = 1, …, 9}, leading to 9 models. The optimal spike scale *s*_0_ was select by the 10-fold 10-time cross-validation according to the CVPL. Using the optimal *s*_0_, we performed further real data analysis. For comparison, we also analyzed the data using the several existing methods as described in the simulation studies.

We performed 10-fold cross-validation with 10 replicates to evaluate the predictive values of the several models. Table 6 summarizes the measures of the prognostic performance on these three data sets, by only using the genes included in pathway. For all the data sets the proposed group spike-and-slab lasso Cox model performed better than the other methods. The above results used only genes mapped in pathways. Additional file 9 shows the measures of the performance on these three data sets, by using the all genes. The genes which were not mapped into any pathway were put together as an additional group. We can see that the prediction performance of the proposed method were still better than the other methods.

**Table 6:** The measures of optimal group spike-and-slab lasso (gsslasso) cox and the lasso cox models for TCGA ovarian cancer, lung adenocarcinoma (LUAD) and breast cancer dataset with pathway genes by 10 times 10-fold cross validation

	Pathway number	Genes included	Methods	CVPL	C-index	Number of non-zero gene
TCGA	271	4260	gsslasso	− 1041.218 (2.118)	0.577 (0.012)	33
ovarian			lasso	− 1042.905 (1.687)	0.533 (0.027)	15
cancer			grlasso	− 1044.110 (12.741)	0.504 (0.014)	24
*N* = 304			grMCP	− 1046.965 (8.604)	0.502 (0.007)	24
			grSCAD	−1042.349 (5.339)	0.503 (0.012)	24
			cMCP	− 1043.373 (2.215)	0.532 (0.019)	13
TCGA	274	4266	gsslasso	− 938.973 (1.675)	0.559 (0.010)	64
LUAD			lasso	− 941.383 (3.720)	0.545 (0.019)	13
*N* = 491			grlasso	− 945.605 (8.137)	0.547 (0.023)	111
			grMCP	− 1092.091 (30.477)	0.512 (0.015)	25
			grSCAD	− 940.358 (1.331)	0.538 (0.021)	123
			cMCP	−942.831 (3.301)	0.530 (0.022)	3
TCGA	275	4385	gsslasso	−996.491 (2.131)	0.640 (0.153)	86
Breast			lasso	−1002.046 (5.356)	0.523 (0.027)	2
cancer			grlasso	− 1001.073 (9.641)	0.590 (0.022)	93
*N* = 1082			grMCP	− 1016.864 (25.290)	0.520 (0.019)	12
			grSCAD	− 1005.299 (2.268)	0.522 (0.007)	24
			cMCP	− 1012.587 (44.339)	0.502 (0.012)	1

Note: Values in the parentheses are standard errors. For group spike-and-slab lasso model, the optimal *s*_0_ = 0.03 for three data sets. In TCGA ovarian cancer, we mapped 4260 genes into 271 pathways. The analyses was performed on these genes including in these pathways. The same is true for other two datasets

The pathway enrichment analyses for these detected genes were summarized in Additional file 10: S4-S6. Additional file 11: S7 presents the genes detected by the proposed and existed methods. Their standardized effects size were also plotted for the three real data sets. There were many common gene among different methods. For ovarian cancer dataset, the genes CYP2R1 and HLA-DOB detected by the proposed gsslasso method, were also detected by both lasso and cMCP methods. For Lung cancer dataset, several genes detected by the proposed gsslasso method, such as VDAC1, EHHADH, ACAT2, KIT, CCND1, PIK3R1, NRAS, GNPNAT1, and KYNU, were also detected by other method. For, breast cancer dataset, two genes HSPA1A and ABCB5 detected by the proposed gsslasso method were also detected by other method.

We found that most of the genes detected by the proposed method were associated with cancers in previous studies. HABP2, detected in ovarian cancer, was associated with familial nonmedullary thyroid cancer [47]. CYP24A1, the main enzyme responsible for the degradation of active vitamin D, plays an important role in many cancer related cellular processes. The associations between CYP24A1 polymorphisms and cancer susceptibility had been evaluated by many studies [48]. Keratin 8 (KRT8) plays an essential role in the development and metastasis of multiple human cancers. A recent study suggested that in clear cell renal cell carcinoma and gastric cancer, KRT8 upregulation promotes tumor metastasis and associated with poor prognosis [49, 50]. E2F7, detected in lung cancer, involved in several cancer studies, which might act as an independent prognostic factor for breast cancer, and Squamous Cell Carcinoma, and gliomas [51–53]. Most of the genes detected by the proposed method in the three real datasets had been found associated with different cancers. These results may provide some interesting information for further studies.

## Discussion

The group structures among various features arise naturally in many biological and medical researches, especially in large-scale omics data. Such grouping information is biologically meaningful and intrinsically encoded in the biological data. Thus it is desirable to incorporate the grouping information into data analysis. Various penalization methods have been designed for such situations [13, 14, 30, 54, 55]. Recently, we have developed a novel hierarchical modeling approach, the group spike-and-slab lasso GLMs, to integrate the variable group information for gene detection and prognostic prediction [27]. In this study, we extended the method to Cox proportional hazards model for analyzing censored survival data.

Similar to the group spike-and-slab lasso GLMs, the key to our group spike-and-slab lasso Cox is the group spike-and-slab double-exponential prior. This prior has significant advantage in variable selection and parameter estimation. It induces weak shrinkage on larger coefficients and strong shrinkage on irrelevant coefficients. In contrast, other methods usually gave a strong shrinkage amount on all the coefficients and resulted in the solutions that non-zero coefficients were shrunk and underestimated. The proposed group spike-and-slab prior allows the model to incorporate the biological similarity of genes within a same pathway into the analysis.

The spike-and-slab prior depends on the spike and slab scale parameters. Our previous study suggested that slab scale *s*_1_ had little influence on model fitting, while the spike scale *s*_0_ strongly affected model performance [25, 26]. A slab scale *s*_1_ value introducing weak shrinkage amount would be helpful to include relevant variables into the model. Therefore, we set *s*_1_ = 1 in our analysis. We evaluated the performance of the proposed model on a grid of values of spike scale *s*_0_ from a reasonable range, e.g., (0, 0.1), and then selecting an optimal value using cross-validation. This is a path-following strategy for fast dynamic posterior exploration of the proposed models, which is similar to the approach of Ročková and George [24, 56]. Additional file 12: Figure S8 a and b show the solution paths under Scenario 2, for the proposed model and the lasso Cox model. Additional file 12: Figure S8 c and d show the profiles of cross-validated palatial log-likelihood by 10-fold cross-validation for the proposed model. These profiles would help to choose optimal tuning parameters. It could be found that, similar to the lasso, the spike-and-slab lasso Cox is a path-following strategy for fast dynamic posterior exploration. However, the solution path is essentially different from that of the lasso model. For the lasso cox model, the number of non-zero coefficient could be a few, even zero if a strong penalty is adopted. However, in the spike-and-slab lasso Cox model, larger coefficients will be always included in the model with weak shrinkage, while irrelevant coefficients are removed (grey path in Additional file 12: Figure S8 a).

Another feature of the proposed spike-and-slab prior is bi-level selection, which is capable of selecting important groups as well as important individual variables within those groups. Several methods perform bi-level selection, including cMCP method [16], SGL [14], and group exponential lasso [18]. The underlying assumption is that the model is sparse at both the group and individual variable levels. The proposed group spike-and-slab lasso Cox model can efficiently perform bi-level selection. In group level, the importance of a group is controlled by the group-specific probability *θ*_*g*_. Within a group, the spike-and-slab prior allows to perform variable selection by shrinking irrelevant or small effect coefficients exactly to zero, without affecting the prediction performance.

The extensive simulation studies show that the prediction performance of the proposed method is always slightly better than cMCP method, and significantly better than all other methods under different scenarios. In the real data analysis, the prediction accuracy of the proposed method incorporating pathway information was slightly improved compared with the existing methods. This might be mainly due to the complex genetic components involved in the expression data, like haplotype blocks, subnetworks, and interaction among the genes. The present model under the linear assumption may not capture these complexities. More sophisticated strategies could potentially enhance prediction accuracy and further improve the models, by defining more precise biological grouping information.

There are several further extensions of the proposed method. For example, it can also be extended to incorporate multiple level group structure, like three-level group structure, i.e. SNP-gene-pathway. In addition, the proposed model takes the spike-and-slab mixture double-exponential prior. Other priors with a spike at zero and includes heavier tails could be investigated, like Cauchy distribution, a special case of Student-t distribution. The theoretical and empirical properties of other priors are different, which may introduce more interesting results.

## Conclusion

Incorporating biological group structure in high-dimensional molecular data analysis can improve the accuracy of disease prediction and power of gene detection. We propose a new hierarchical Cox model, gsslasso Cox, for incorporating biological pathway information for predicting disease survival outcomes and detecting associated genes. We develop a fast and stable deterministic algorithm to fit the proposed models. Extensive simulation studies and real applications show that compared with several existing methods, the proposed approach provides more accurate parameter estimation and survival prediction. The proposed method has been implemented in a freely available R package BhGLM.

## Additional files

Additional file 1: **Figure S1.** The parameter estimation averaged over 100 replicates for the group spike-and-slab lasso Cox (gsslasso), the lasso, grlasso, grMCP, grSCAD, and cMCP methods for Scenario 2. Blue cycles denote the simulated non-zero values. Black points and lines represent the estimated values and the interval estimates of coefficients. (PDF 523 kb)

Additional file 2: **Figure S2.** The parameter estimation averaged over 100 replicates for the group spike-and-slab lasso Cox (gsslasso), the lasso and grlasso methods for Scenario 3. Blue cycles denote the simulated non-zero values. Black points and lines represent the estimated values and the interval estimates of coefficients. The main title of each plot denotes the varying group size for scenario 3. (PDF 778 kb)

Additional file 3: **Figure S3.** The parameter estimation averaged over 100 replicates for grMCP, grSCAD, and cMCP methods for Scenario 3. Blue cycles denote the simulated non-zero values. Black points and lines represent the estimated values and the interval estimates of coefficients. The main title of each plot denotes the varying group size for Scenario 3. (PDF 767 kb)

Additional file 4: **Figure S4.** The parameter estimation averaged over 100 replicates for the group spike-and-slab lasso Cox (gsslasso), the lasso and grlasso methods for Scenario 4. Blue cycles denote the simulated non-zero values. Black points and lines represent the estimated values and the interval estimates of coefficients. The main title of each plot denotes the varying the number of non-null group for Scenario 4. (PDF 791 kb)

Additional file 5: **Figure S5.** The parameter estimation averaged over 100 replicates for grMCP, grSCAD, and cMCP for Scenario 4. Blue cycles denote the simulated non-zero values. Black points and lines represent the estimated values and the interval estimates of coefficients. The main title of each plot denotes the varying the number of non-null group for Scenario 4. (PDF 783 kb)

Additional file 6: **Figure S6**. The parameter estimation averaged over 100 replicates for the group spike-and-slab lasso Cox (gsslasso), the lasso and grlasso methods for Scenario 5. Blue cycles denote the simulated non-zero values. Black points and lines represent the estimated values and the interval estimates of coefficients. The main title of each plot denotes the varying the number of non-null group for Scenario 5. (PDF 1054 kb)

Additional file 7: **Figure S7.** The parameter estimation averaged over 100 replicates for grMCP, grSCAD, and cMCP for Scenario 5. Blue cycles denote the simulated non-zero values. Black points and lines represent the estimated values and the interval estimates of coefficients. The main title of each plot denotes the varying the number of non-null group for Scenario 5. (PDF 778 kb)

Additional file 8: **S1, S2, and S3.** The detailed information of genes shared by different pathways for ovarian cancer, lung cancer and breast cancer, respectively. (ZIP 213 kb)

Additional file 9: **Table S1.** The measures of optimal group spike-and-slab lasso (gsslasso) cox and the lasso cox models for TCGA ovarian cancer, lung adenocarcinoma (LUAD) and breast cancer dataset with all genes by 10 times 10-fold cross validation. (DOCX 20 kb)

Additional file 10: **S4, S5 and S6.** The pathway enrichment analyses for these detected genes for ovarian cancer, lung cancer and breast cancer, respectively. (ZIP 15 kb)

Additional file 11: **S7.** The detected genes and their standardized effect sizes estimated by the group spike-and-slab lasso Cox model and five existed methods for TCGA real datasets. (PDF 1340 kb)

Additional file 12: **Figure S8.** The solution path and cross-validated partial loglikelihood profiles of the group spike-and-slab lasso Cox (a, c) and the lasso (b, d) based on the Scenario 2. The colored points on the solution path represent the estimated values of assumed eight non-zero coefficients, and the circles represent true non-zero coefficients. Vertical lines correspond to the optimal models. (PDF 1052 kb)

## Abbreviations

cMCP: Composite minimax concave penalty
CVPL: Cross-validated partial likelihood
grlasso: Group lasso
grMCP: Group minimax concave penalty
grSCAD: Group smoothly clipped absolute deviation
gsslasso cox: Group spike-and-slab lasso cox model
lasso: Least absolute shrinkage and selection operator
