# Supplementary figures and images for: Gsslasso Cox: a Bayesian hierarchical model for predicting survival and detecting associated genes by incorporating pathway information
Source: BMC Bioinformatics. 2019 Feb 27;20:94. doi: 10.1186/s12859-019-2656-1 (PMC6391807; doi:10.1186/s12859-019-2656-1)

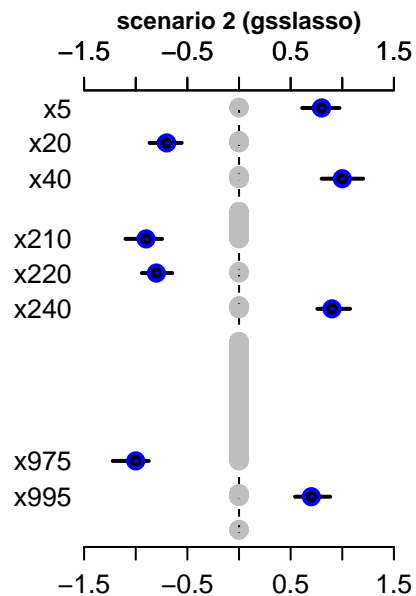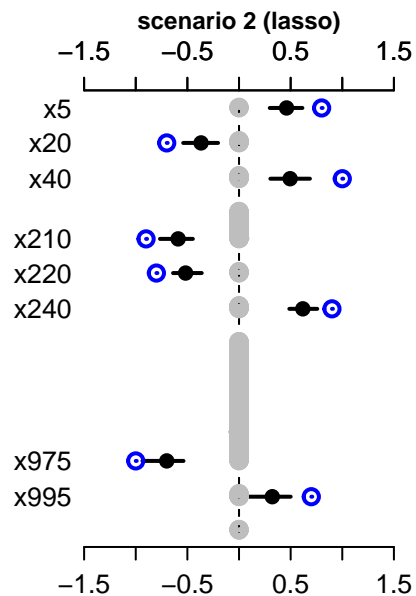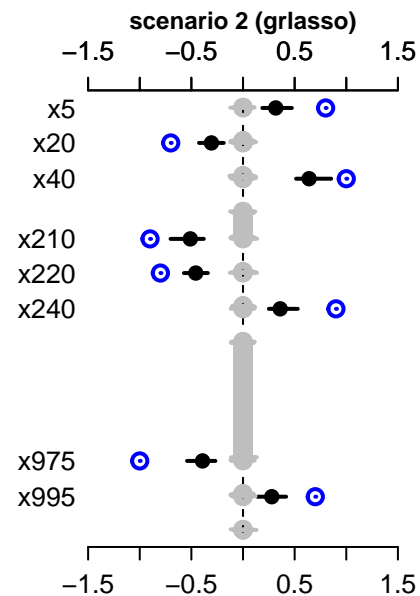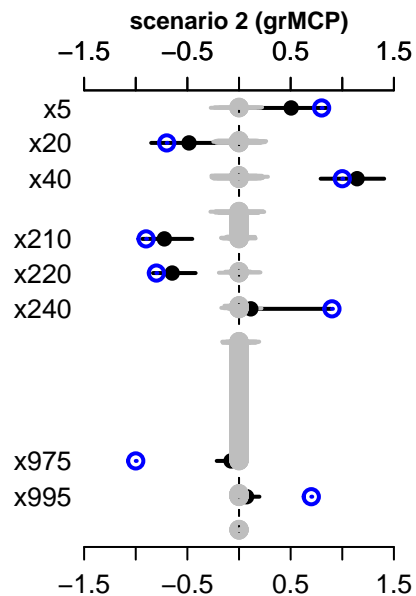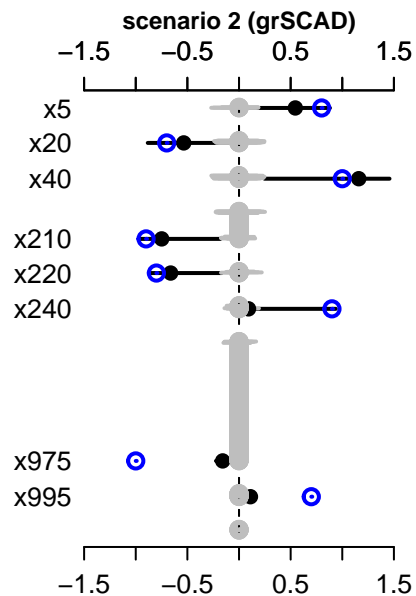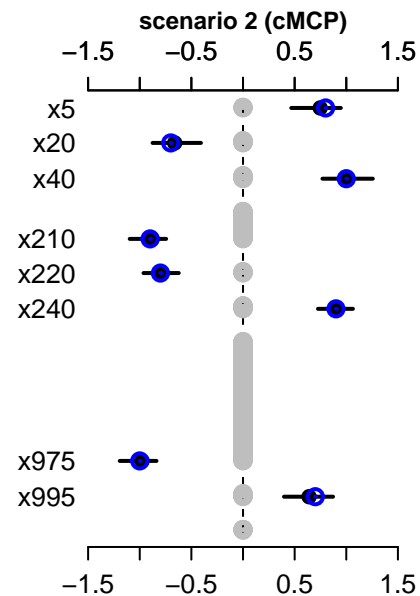

Supplement: Supplementary file 1 — Figure S1. The parameter estimation averaged over 100 replicates for the group spike-and-slab lasso Cox (gsslasso), the lasso, grlasso, grMCP, grSCAD, and cMCP methods for Scenario 2. Blue cycles denote the simulated non-zero values. Black points and lines represent the estimated values and the interval estimates of coefficients. (PDF 523 kb) [file 12859_2019_2656_MOESM1_ESM.pdf]

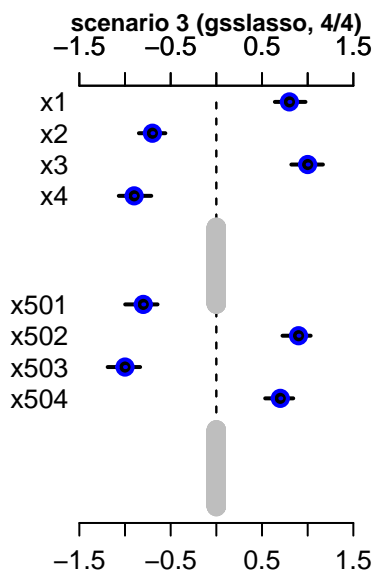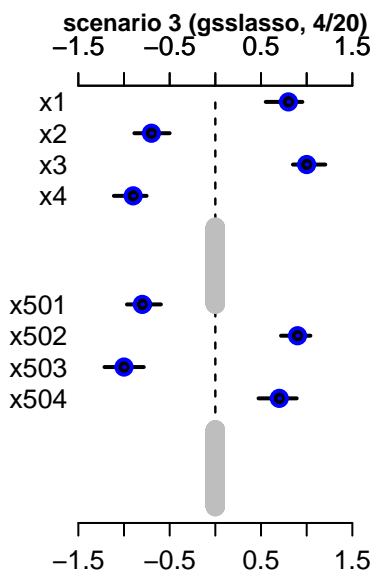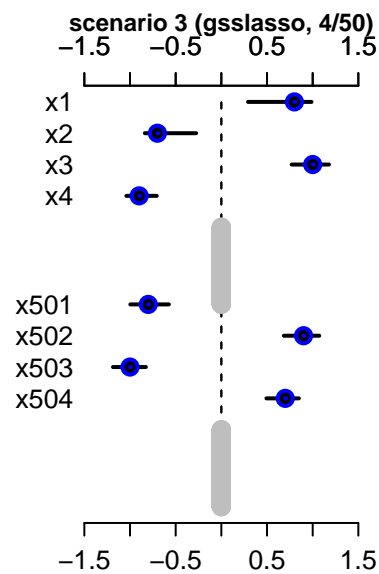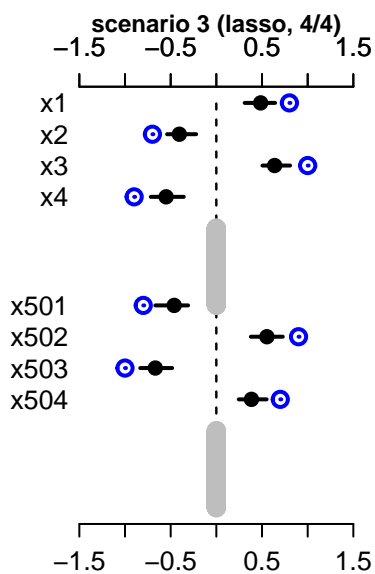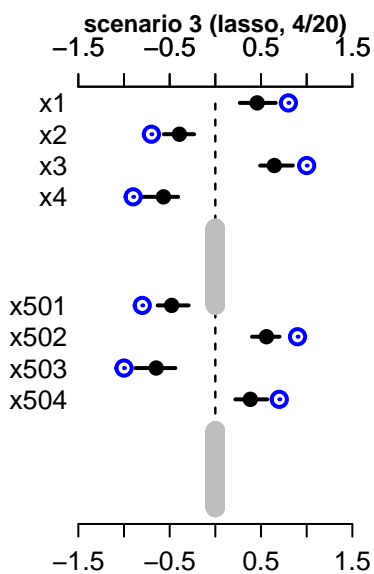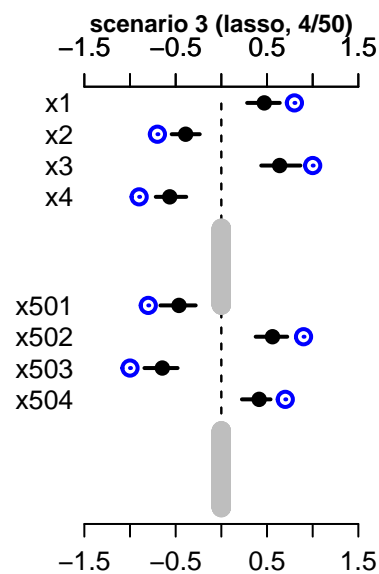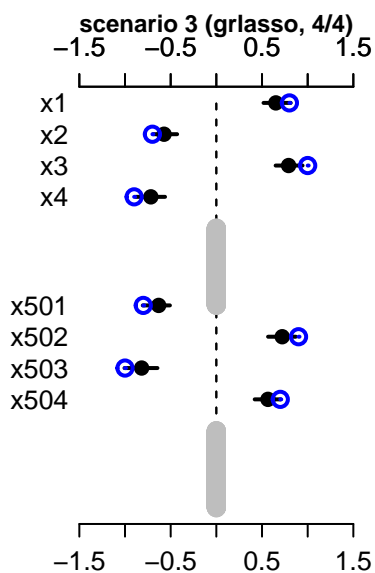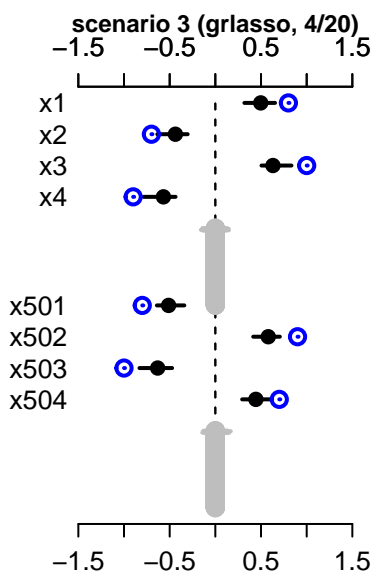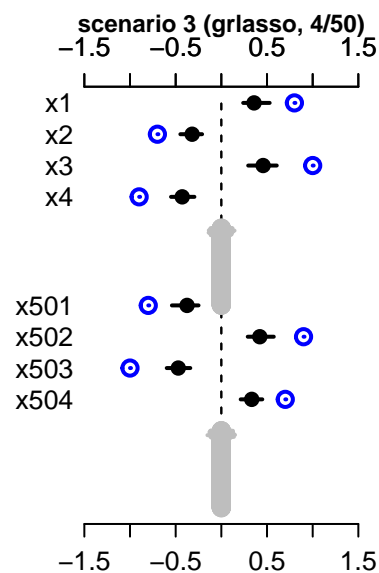

Supplement: Supplementary file 2 — Figure S2. The parameter estimation averaged over 100 replicates for the group spike-and-slab lasso Cox (gsslasso), the lasso and grlasso methods for Scenario 3. Blue cycles denote the simulated non-zero values. Black points and lines represent the estimated values and the interval estimates of coefficients. The main title of each plot denotes the varying group size for scenario 3. (PDF 778 kb) [file 12859_2019_2656_MOESM2_ESM.pdf]

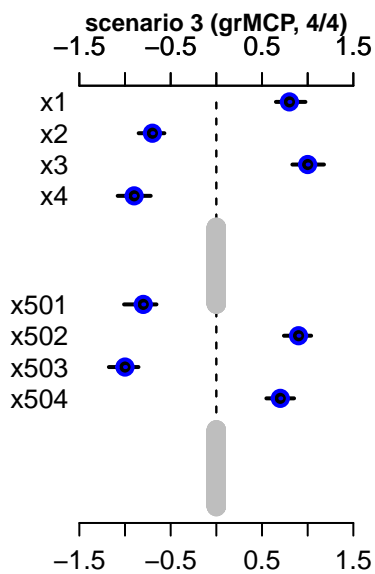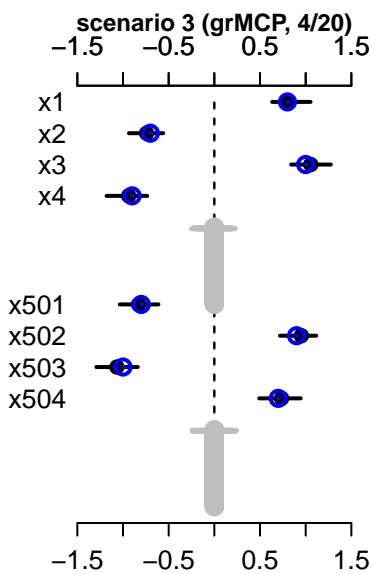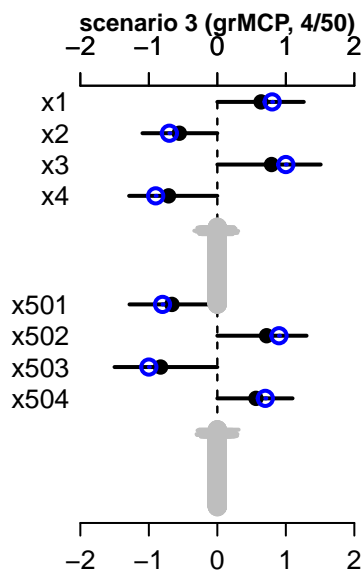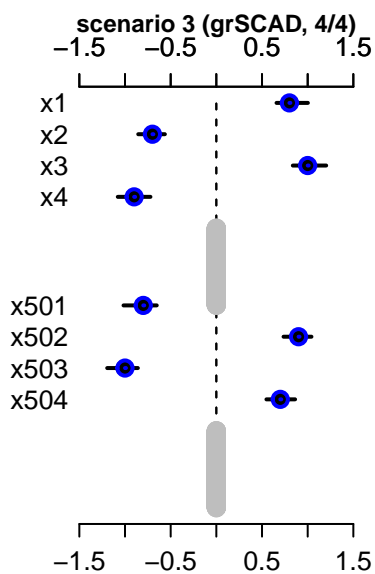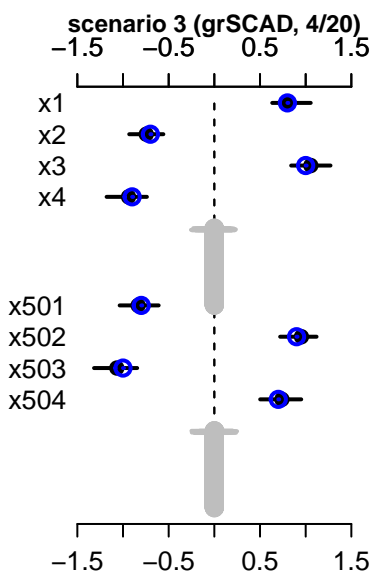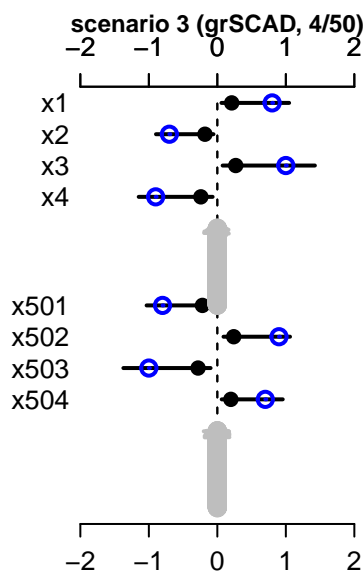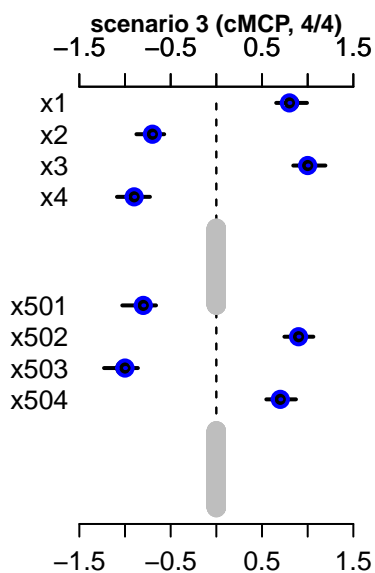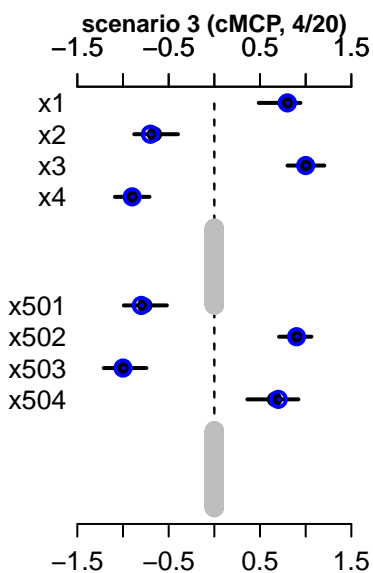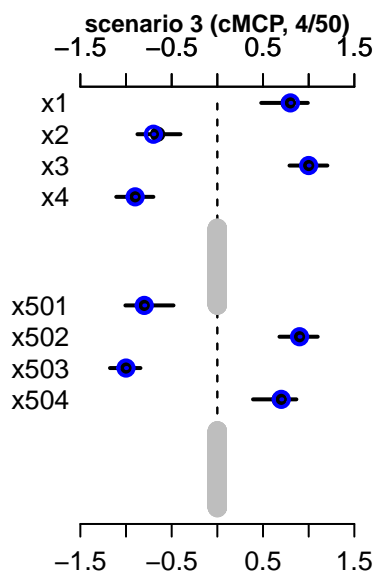

Supplement: Supplementary file 3 — Figure S3. The parameter estimation averaged over 100 replicates for grMCP, grSCAD, and cMCP methods for Scenario 3. Blue cycles denote the simulated non-zero values. Black points and lines represent the estimated values and the interval estimates of coefficients. The main title of each plot denotes the varying group size for Scenario 3. (PDF 767 kb) [file 12859_2019_2656_MOESM3_ESM.pdf]

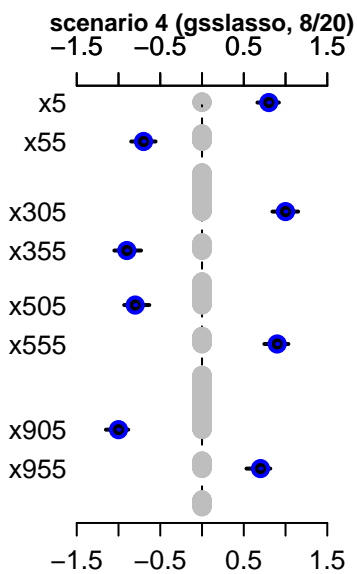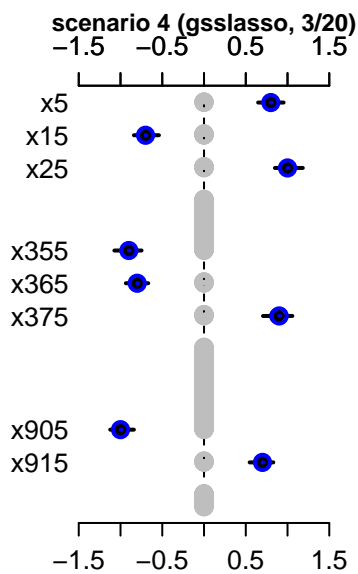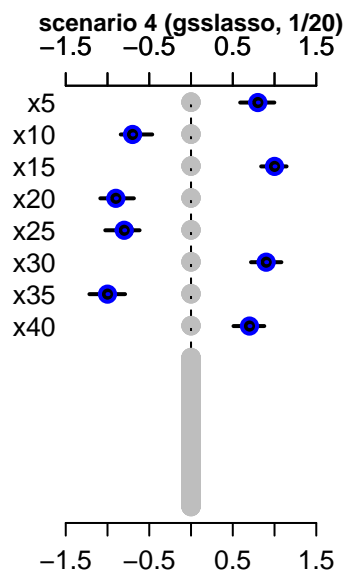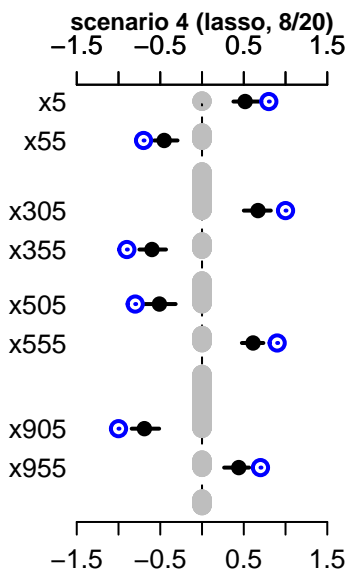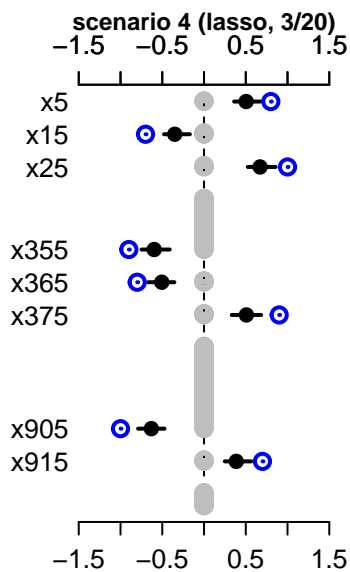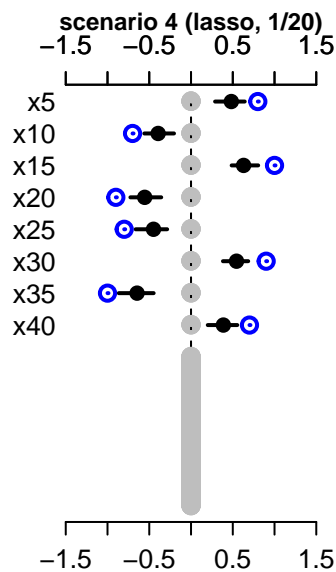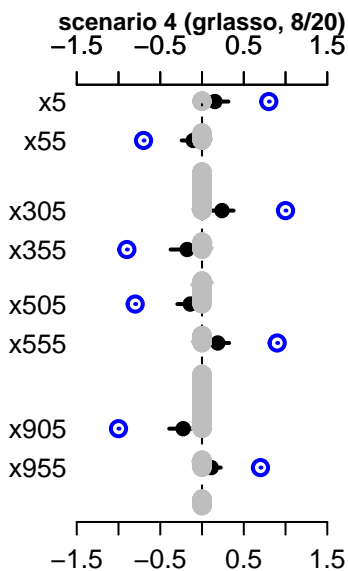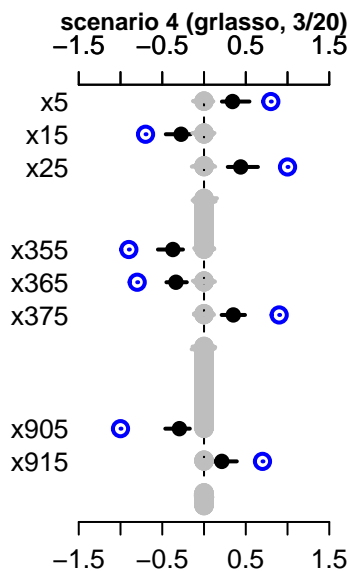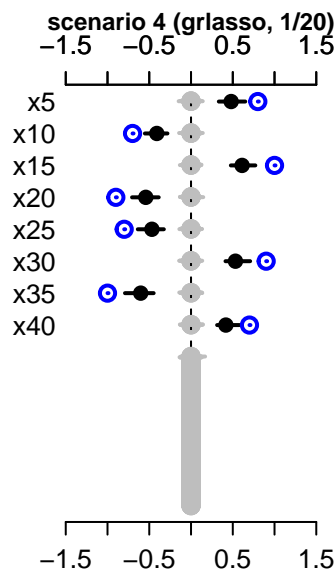

Supplement: Supplementary file 4 — Figure S4. The parameter estimation averaged over 100 replicates for the group spike-and-slab lasso Cox (gsslasso), the lasso and grlasso methods for Scenario 4. Blue cycles denote the simulated non-zero values. Black points and lines represent the estimated values and the interval estimates of coefficients. The main title of each plot denotes the varying the number of non-null group for Scenario 4. (PDF 791 kb) [file 12859_2019_2656_MOESM4_ESM.pdf]

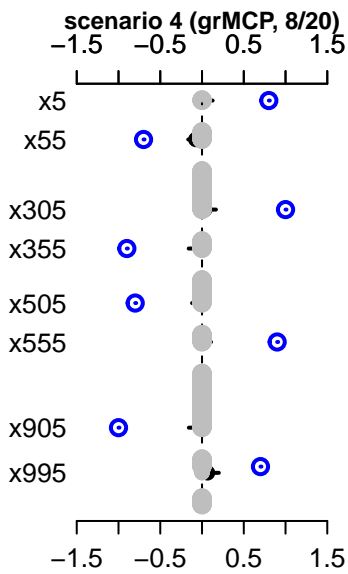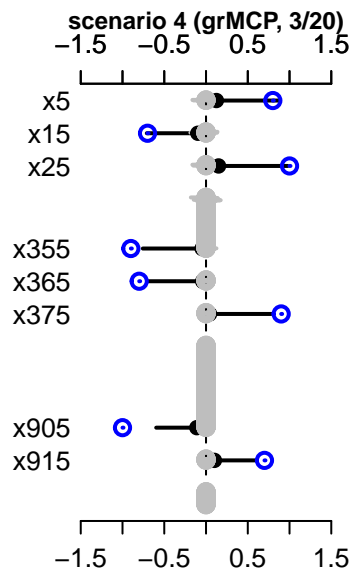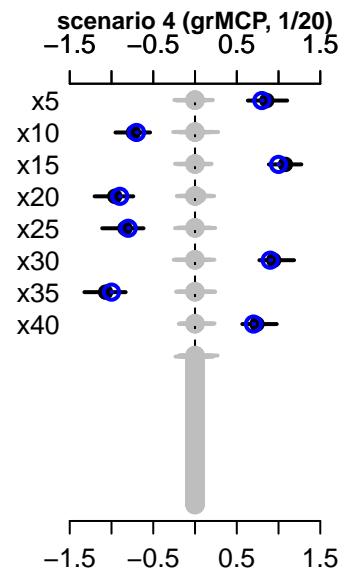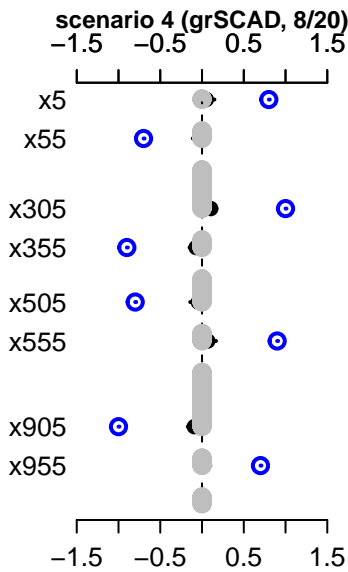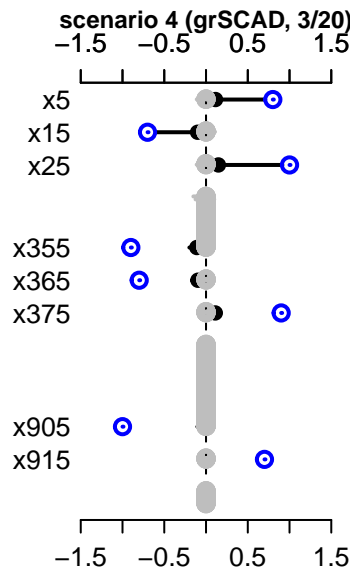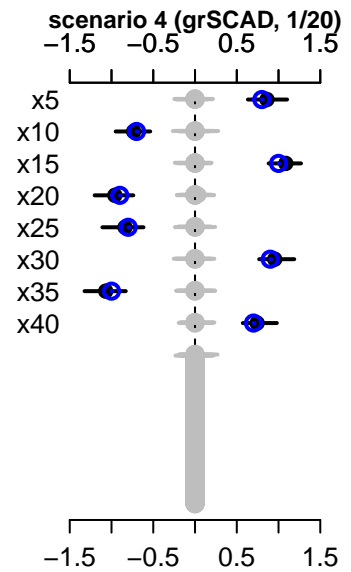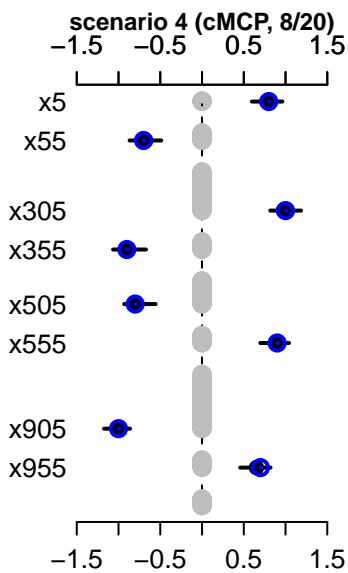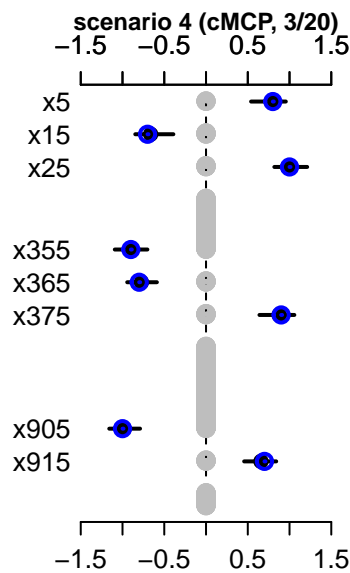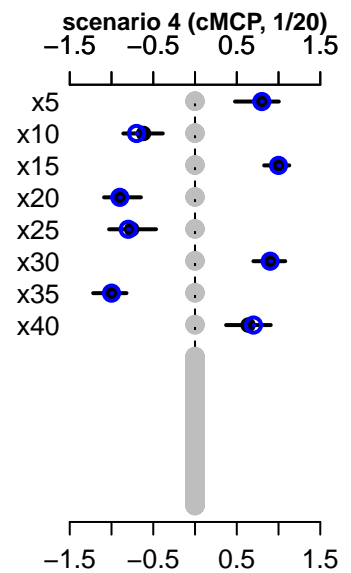

Supplement: Supplementary file 5 — Figure S5. The parameter estimation averaged over 100 replicates for grMCP, grSCAD, and cMCP for Scenario 4. Blue cycles denote the simulated non-zero values. Black points and lines represent the estimated values and the interval estimates of coefficients. The main title of each plot denotes the varying the number of non-null group for Scenario 4. (PDF 783 kb) [file 12859_2019_2656_MOESM5_ESM.pdf]

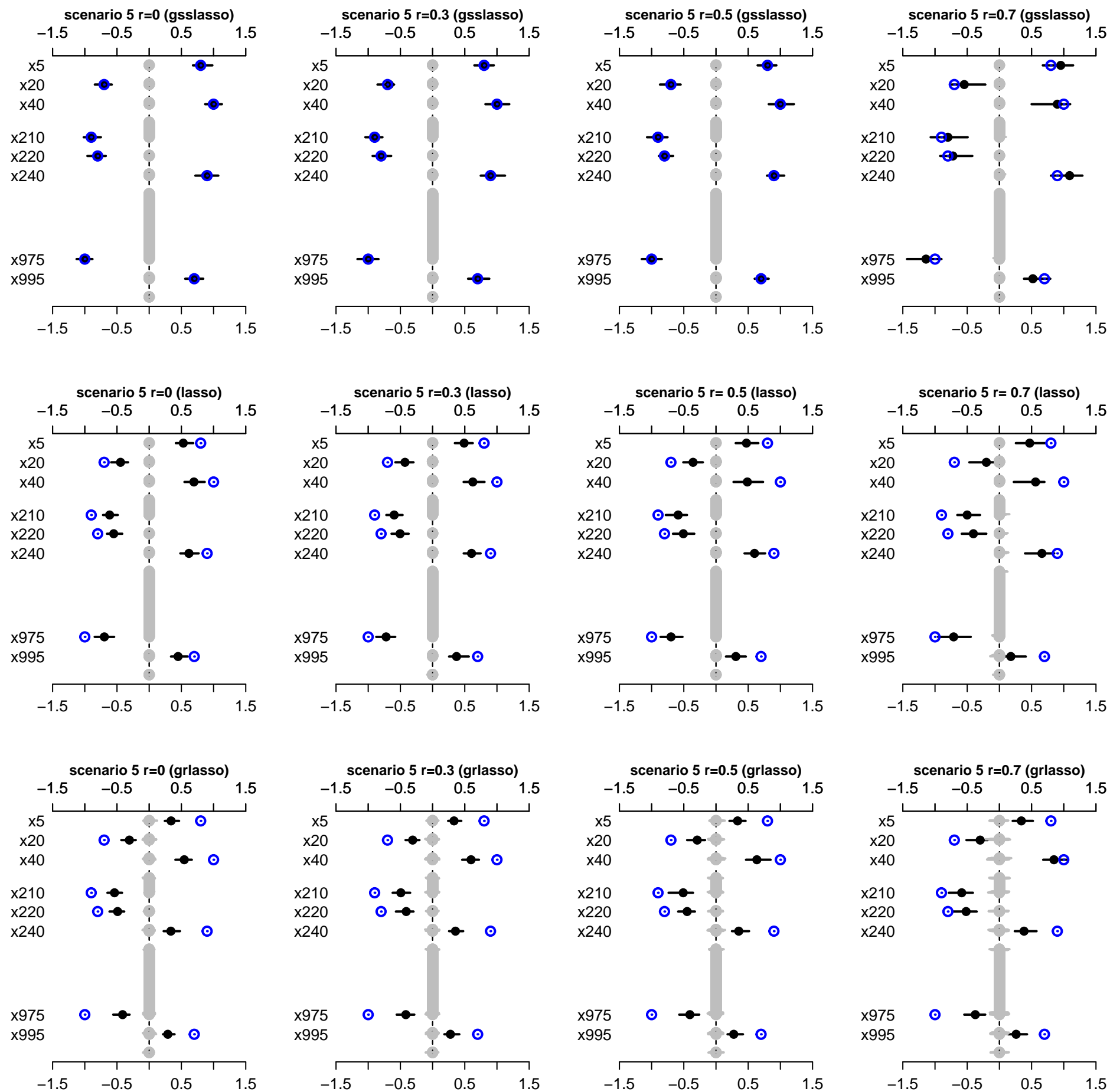

Supplement: Supplementary file 6 — Figure S6. The parameter estimation averaged over 100 replicates for the group spike-and-slab lasso Cox (gsslasso), the lasso and grlasso methods for Scenario 5. Blue cycles denote the simulated non-zero values. Black points and lines represent the estimated values and the interval estimates of coefficients. The main title of each plot denotes the varying the number of non-null group for Scenario 5. (PDF 1054 kb) [file 12859_2019_2656_MOESM6_ESM.pdf]

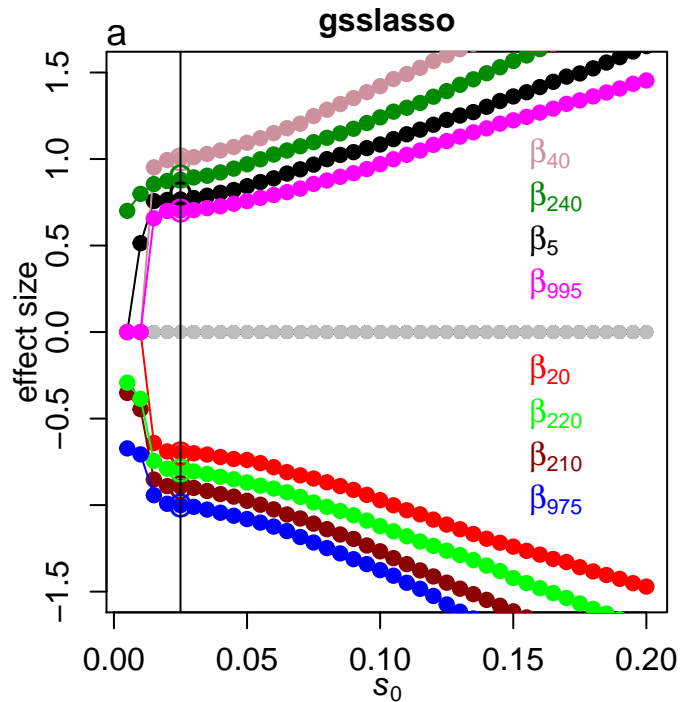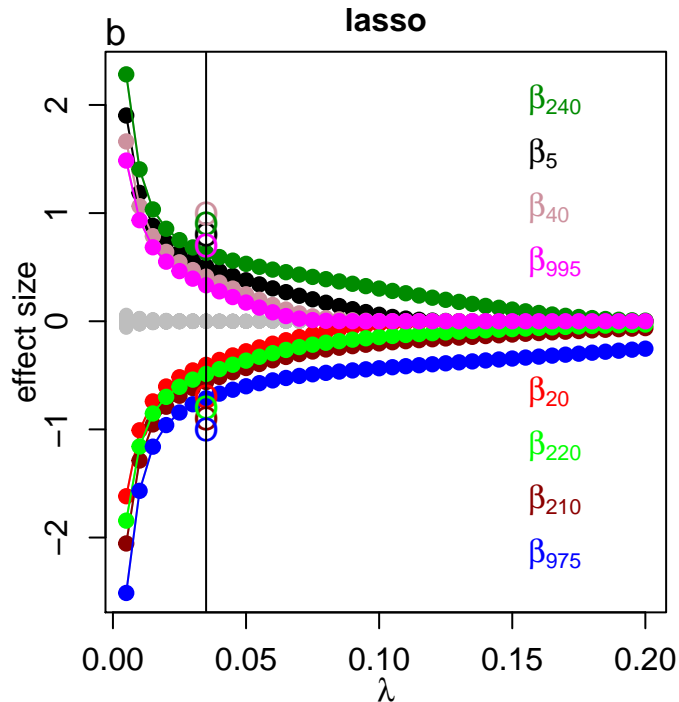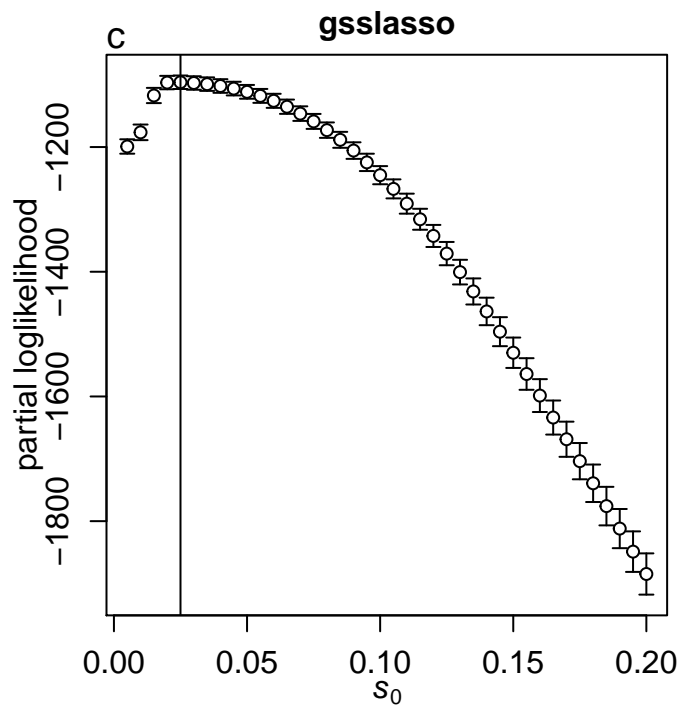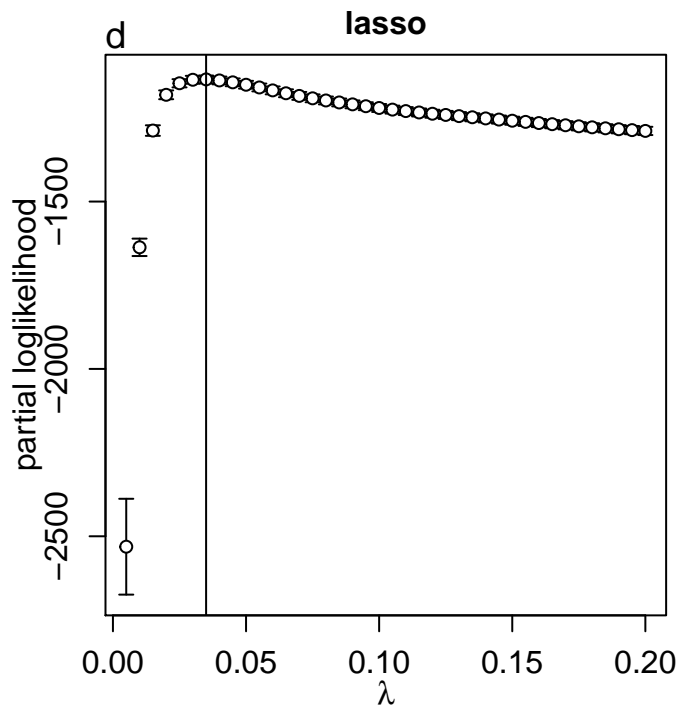

Supplement: Supplementary file 7 — Figure S7. The parameter estimation averaged over 100 replicates for grMCP, grSCAD, and cMCP for Scenario 5. Blue cycles denote the simulated non-zero values. Black points and lines represent the estimated values and the interval estimates of coefficients. The main title of each plot denotes the varying the number of non-null group for Scenario 5. (PDF 778 kb) [file 12859_2019_2656_MOESM7_ESM.pdf]

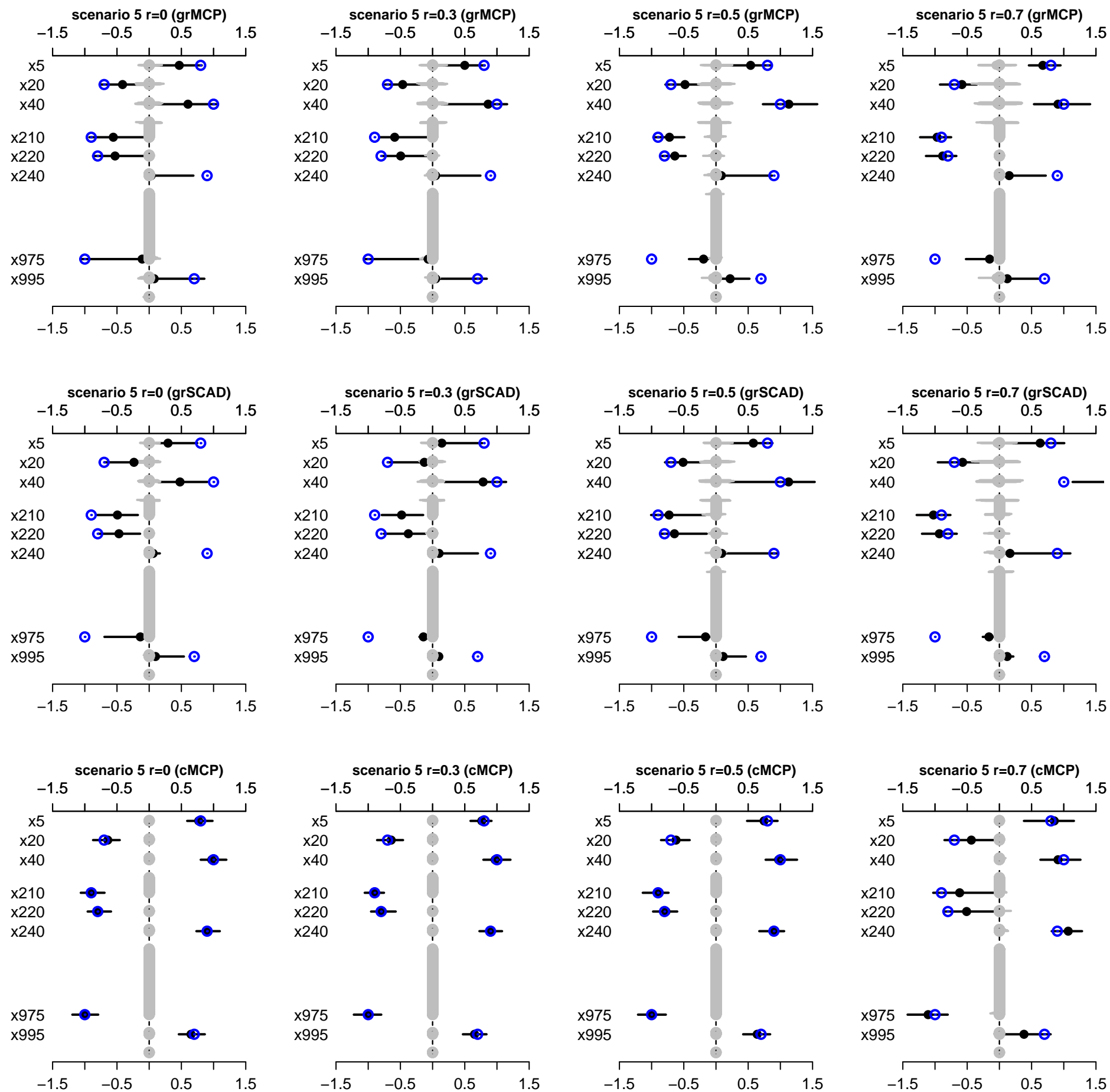

Supplement: Supplementary file 12 — Figure S8. The solution path and cross-validated partial loglikelihood profiles of the group spike-and-slab lasso Cox (a, c) and the lasso (b, d) based on the Scenario 2. The colored points on the solution path represent the estimated values of assumed eight non-zero coefficients, and the circles represent true non-zero coefficients. Vertical lines correspond to the optimal models. (PDF 1052 kb) [file 12859_2019_2656_MOESM12_ESM.pdf]
