# Supplementary material for: Gsslasso Cox: a Bayesian hierarchical model for predicting survival and detecting associated genes by incorporating pathway information
Source: BMC Bioinformatics. 2019 Feb 27;20:94. doi: 10.1186/s12859-019-2656-1 (PMC6391807; doi:10.1186/s12859-019-2656-1)
Supplement: Supplementary file 9 — Table S1. The measures of optimal group spike-and-slab lasso (gsslasso) cox and the lasso cox models for TCGA ovarian cancer, lung adenocarcinoma (LUAD) and breast cancer dataset with all genes by 10 times 10-fold cross validation. (DOCX 20 kb) [file 12859_2019_2656_MOESM9_ESM.docx]

**Table S1. The measures of optimal group spike-and-slab lasso (gsslasso) cox and the lasso cox models for TCGA ovarian cancer, lung adenocarcinoma (LUAD) and breast cancer dataset with all genes by 10 times 10-fold cross validation.**

|  | Pathway  number | Genes  included | Methods | CVPL | C-index | Number of  non-zero gene |
| --- | --- | --- | --- | --- | --- | --- |
| TCGA | 271+1 | 14265 | Gsslasso(0.01) | -1031.150(0.471) | 0.572(0.002) | 3 |
| ovarian |  |  | lasso | -1041.348(1.169) | 0.539(0.017) | 18 |
| cancer |  |  | grlasso | -1044.777(9.036) | 0.504(0.009) | 24 |
| n=304 |  |  | grMCP | -1040.971(4.631) | 0.501(0.009) | 24 |
|  |  |  | grSCAD | -1048.933(13.065) | 0.505(0.010) | 24 |
|  |  |  | cMCP | -1044.971(2.243) | 0.518(0.016) | 2 |
| TCGA | 274+1 | 14143 | Gsslasso(0.02) | -940.9470(0.760) | 0.532(0.019) | 6 |
| LUAD |  |  | lasso | -939.568(2.512) | 0.549(0.020) | 20 |
| n=491 |  |  | grlasso | -948.202(14.523) | 0.558(0.013) | 123 |
|  |  |  | grMCP | -1015.603(129.818) | 0.512(0.027) | 25 |
|  |  |  | grSCAD | -941.580(2.590) | 0.544(0.021) | 41 |
|  |  |  | cMCP | -942.206(2.553) | 0.529(0.019) | 9 |
| TCGA | 275+1 | 14077 | Gsslasso(0.01) | -986.253(0.617) | 0.578(0.004) | 32 |
| Breast |  |  | lasso | -997.860(1.868) | 0.522(0.023) | 4 |
| cancer |  |  | grlasso | -1000.398(2.476) | 0.589(0.017) | 65 |
| n=1082 |  |  | grMCP | -1030.787(50.749) | 0.520(0.002) | 22 |
|  |  |  | grSCAD | -1011.599(20.252) | 0.527(0.012) | 31 |
|  |  |  | cMCP | -1025.103(86.795) | 0.507(0.021) | 3 |

Note: Values in the parentheses are standard errors. For group spike-and-slab lasso model, the optimal *s*_0_ = 0.01, 0.02, and 0.01 for ovarian cancer, lung adenocarcinoma, and breast cancer, respectively. In TCGA ovarian cancer, we mapped genes into 271 pathways. The rest genes were put together as an additional group. The same is true for other two datasets. The analyses were performed including all genes.
