# Supplementary material for: Gsslasso Cox: a Bayesian hierarchical model for predicting survival and detecting associated genes by incorporating pathway information
Source: BMC Bioinformatics. 2019 Feb 27;20:94. doi: 10.1186/s12859-019-2656-1 (PMC6391807; doi:10.1186/s12859-019-2656-1)

Results for ovarian cancer by different methods

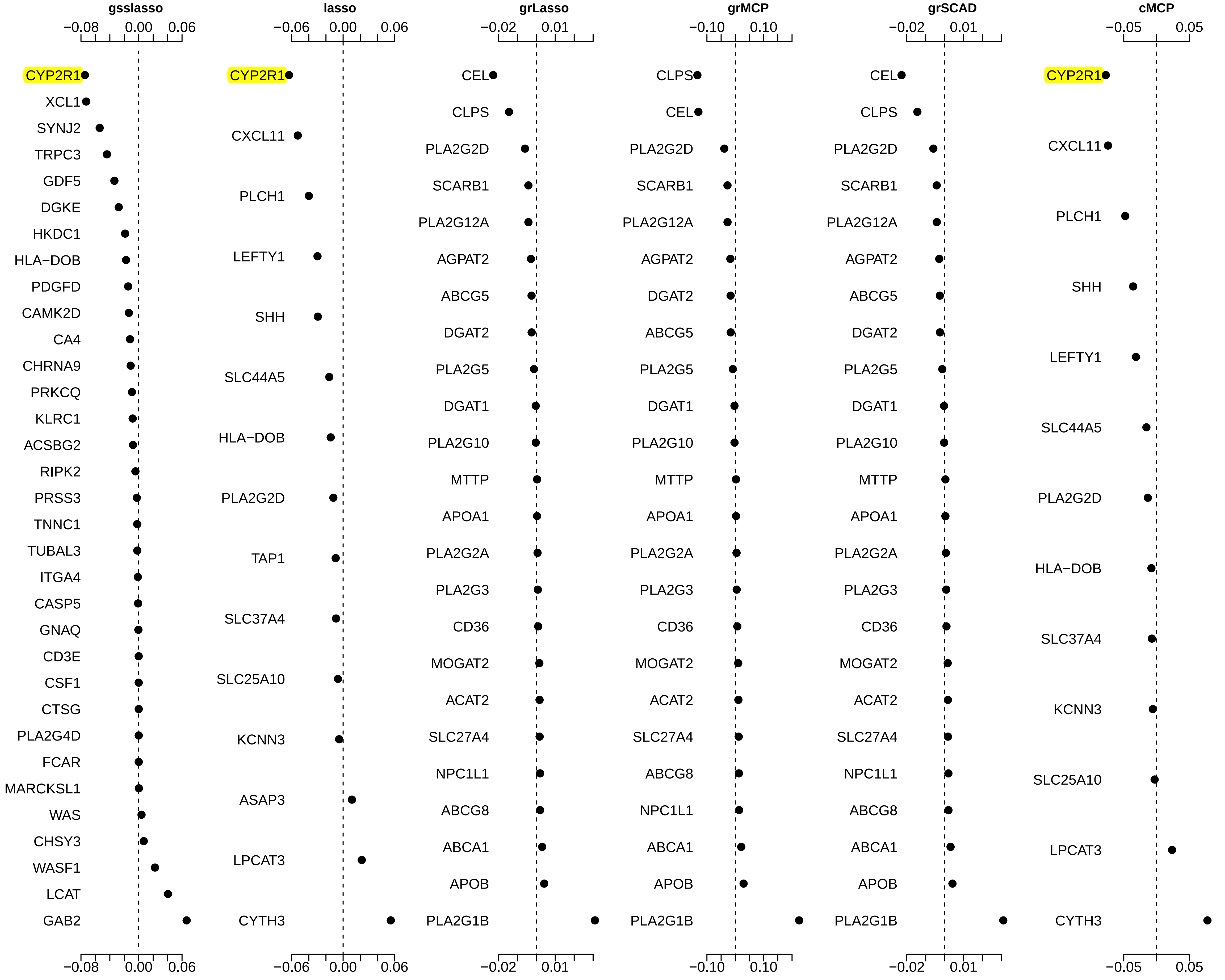

Results for lung cancer by different methods

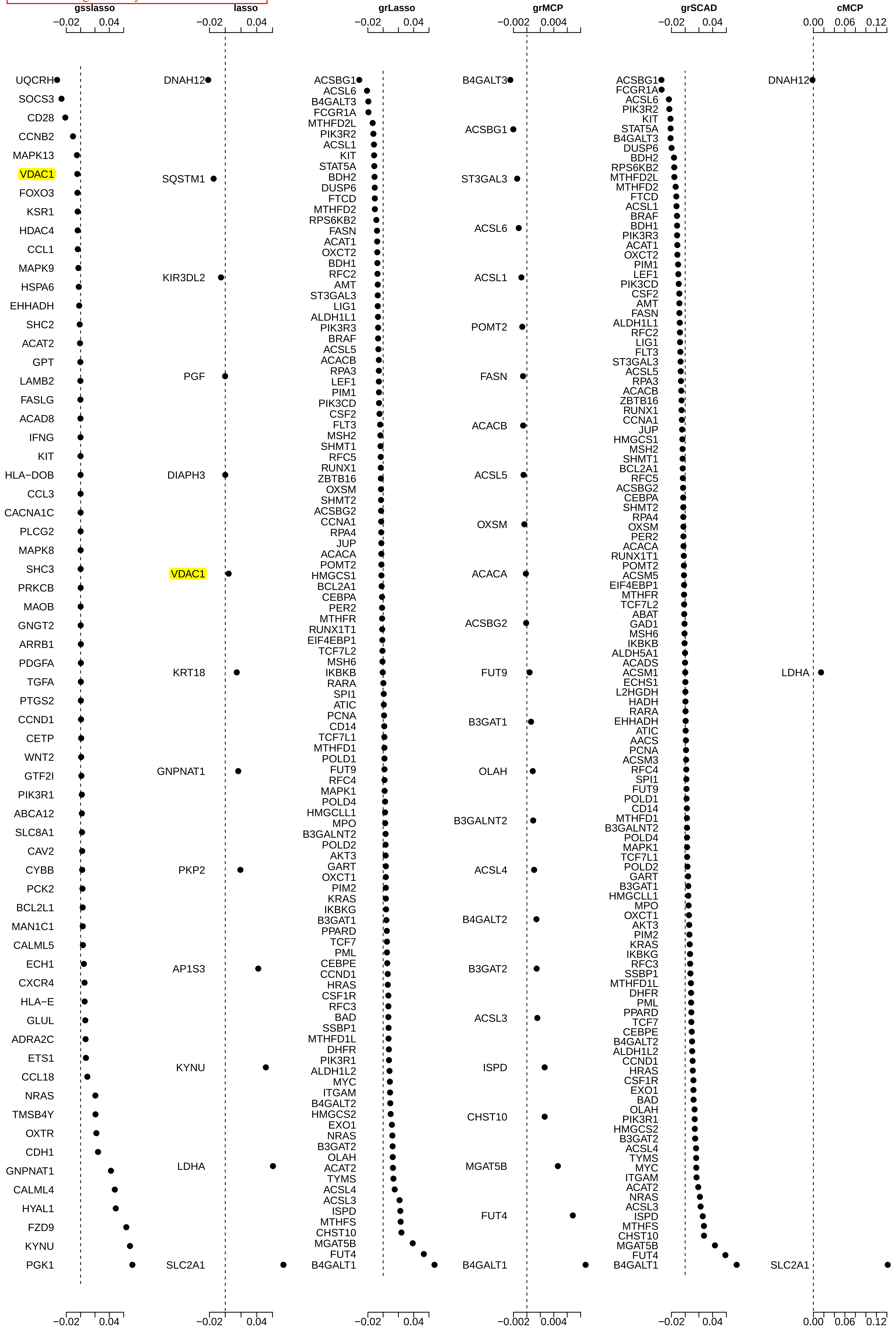

Results for breast cancer by different methods

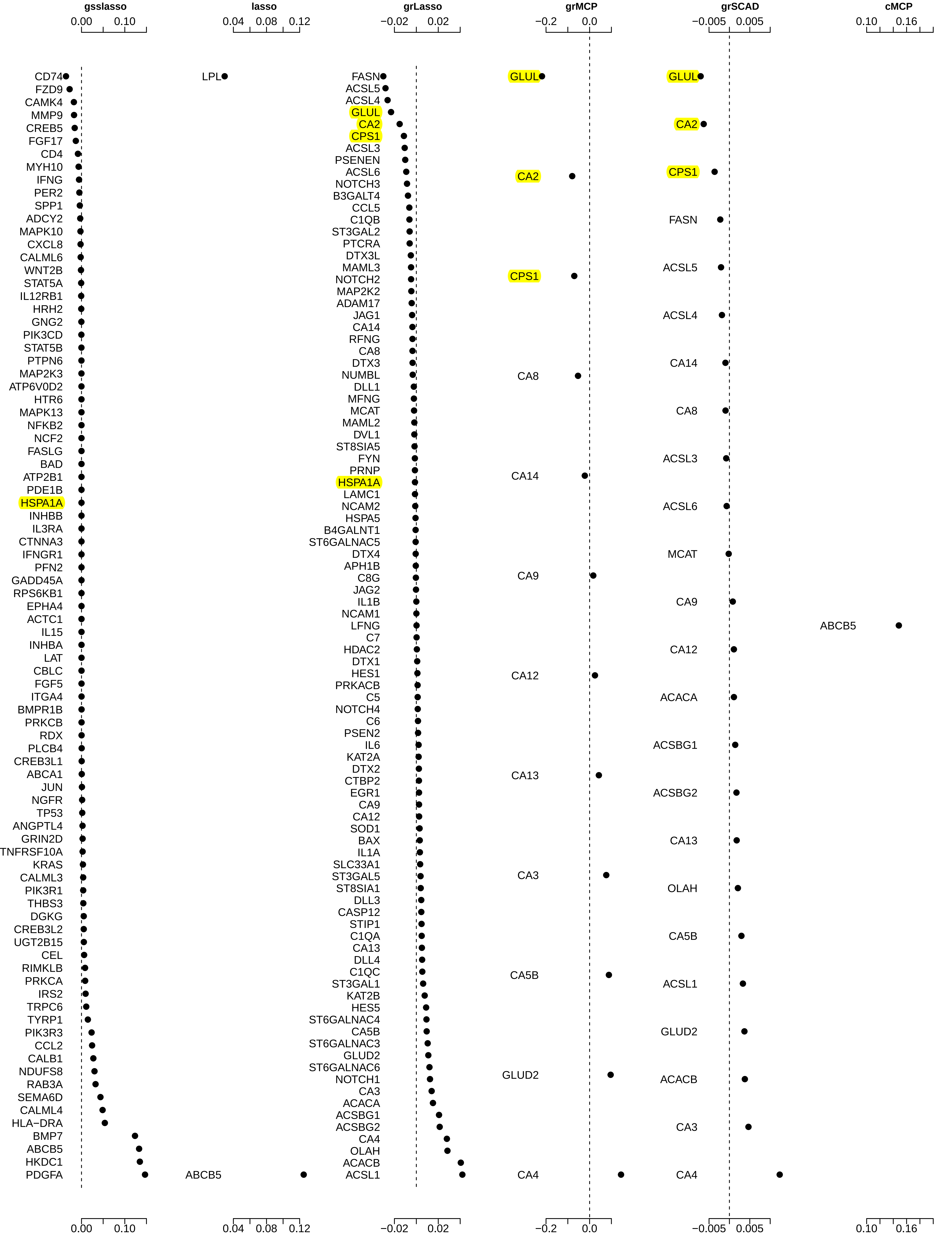

Supplement: Supplementary file 11 — S7. The detected genes and their standardized effect sizes estimated by the group spike-and-slab lasso Cox model and five existed methods for TCGA real datasets. (PDF 1340 kb) [file 12859_2019_2656_MOESM11_ESM.pdf]
